# Quantitation of Vitamin B6 Vitamers and Glycosides in German Alcohol-Free and Full-Strength Beer by a Stable Isotope Dilution LC–MS/MS Method

**DOI:** 10.1021/acs.jafc.5c14229

**Published:** 2026-04-22

**Authors:** Simone Jahner, Elias Geilich, Carina Hagenauer, Michael Rychlik

**Affiliations:** † Chair of Analytical Food Chemistry, Technical University of Munich, D-85365 Freising, Germany; ‡ Centre for Nutrition and Food Sciences, University of Queensland, Brisbane, Queensland 4072, Australia

**Keywords:** beer, alcohol free, pyridoxine, pyridoxine-5′-β-glucoside, stable isotope dilution assay

## Abstract

Vitamin B6 is an essential cofactor of numerous enzymes, and beer may contribute substantially to its dietary intake. While vitamin B6 occurs predominantly as glycosides in plants, data on the complete vitamer profile in beer remain scarce. Here, a novel SIDA LC–MS/MS method was developed for the native quantification of pyridoxine, pyridoxal, pyridoxamine, 4-pyridoxic acid, pyridoxine-5′-β-glucoside, pyridoxine-5′-β-maltoside, and pyridoxine-5-β-cellobioside. The method was validated for linearity, precision, recovery, and limits of quantification (0.20*-*12.9 μg/L) and detection. Analysis of 65 beers revealed total vitamin B6 concentrations ranging from 95.3 to 1020 μg/L, including multiple B6 glycosides. Variations in vitamin B6 composition were primarily associated with raw materials rather than with brewing technology. No significant differences were observed between alcohol-free and corresponding full-strength beers, whereas wheat beers showed vitamin B6 contents significantly lower than those of lager beers. Bock beer exhibited the highest concentrations. This study provides a comprehensive analytical basis for future investigations of vitamin B6 during brewing.

## Graphical abstract

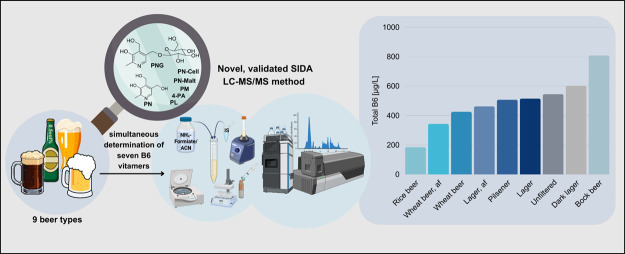

## 1 Introduction

Alcohol-free beer consumption has significantly increased in the past decade, amounting to a volume of 4 mil. hectoliters in Germany alone and 75 mils. hectoliters worldwide in 2024.^1,2^ Alcohol-free beer has long been highlighted for its health-promoting compounds, which are also present in full-strength beer.^3^ However, the adverse effects of alcohol in the latter overshadow the presence of healthy substances. Considering the high prevalence of liver disease due to excessive intake of alcoholic beverages, with a correlation to cancer and noncommunicable health concerns such as social dysfunction, poor health, and reduced productivity, alcohol-free alternatives represent a healthier choice compared to conventional beer.^3^ Recent studies further emphasized ethanol’s genotoxic and carcinogenic properties,^4^ with no threshold level for safe consumption as postulated by the WHO in 2023.^5^ Simultaneously, due to emerging trends in higher-income countries driven by the ambition to adopt a healthier lifestyle, consumers value alcohol-free beer for its isotonic properties and lower calorie content per serving compared to conventional beer, provided that its sensory properties are equally appealing as those of their alcohol-containing counterparts.^6,7^ Especially alcohol-free beer prepared by suppressed fermentation contains many isotonic compounds, such as pre- and probiotics, polyphenols, B-vitamins, and carbohydrates.^8–10^

Among the group of B-vitamins, vitamin B6 with its vitamers pyridoxine (PN), pyridoxal (PL), and pyridoxamine (PM), including their respective 5′-phosphates PN-phosphate (PNP), PL-phosphate (PLP), and PM-phosphate (PMP), plays a crucial role as a cofactor in more than 150 enzymatic reactions, which amounts to approximately 4% of all characterized enzymatic activities.^11–13^ While vitamin B6 is an essential nutrient that humans cannot synthesize, all free vitamer forms absorbed in the diet are interconvertible via the *salvage* pathway in human metabolism to generate the enzymatically active form PLP.^14^ The main reactions are amino acid degradation and biosynthesis, including aldol cleavage, racemization, and transamination, among others. Hence, PLP is necessary for the biosynthesis of neurotransmitters such as dopamine, serotonin, and γ-aminobutyric acid (GABA). Further, it is also involved in fatty acid metabolism (e.g., desaturation of linoleic acid) and in carbohydrate degradation (e.g., the release of glucose from glycogen). Usually, PLP is covalently bound to the ε-lysine group as *a Schiff base* in a B6-dependent enzyme and cleaved on demand to react with the amino group of the substrate.^11,14^ Due to the high number of reactions mediated by PLP, symptoms of deficiency can vary between subjects and are often interwoven with other vitamin B deficiencies, such as vitamin B_12_ or B_9_.^15,16^ Deficiencies of all three groups of B vitamins cause elevated plasma homocysteine levels because of their involvement in the one-carbon metabolism.^16^ Consequently, vitamin B6 deficiency is linked to an increased risk of cardiovascular disease.^17^ In a study on the influence of the type of alcoholic beverage consumed in moderate amounts on blood homocysteine levels, an inverse correlation was observed, even though chronic alcohol abuse is linked with lower PLP plasma levels.^18,19^ Due to the involvement of PLP in the tryptophan-serotonin pathway, B6 deficiency was also correlated with symptoms of depression.^20,21^ Furthermore, a connection between B6 insufficiency and diabetes, inflammation, and cancer has been observed.^13^

In plants, the main share (up to 82%^22^) of B6 is bound as 5′-*O*-β-glucopyranosyl pyridoxine (PNG), which in a balanced omnivorous diet was estimated to provide approximately 15% of the total B6 intake.^23–25^ Although conjugation to disaccharides (e.g., PN-5′-β-cellobioside) and more complex carbohydrates has been described in rice bran, the entire structural diversity of putative B6 glycosides has yet to be unveiled.^26,27^ While all accessible forms of vitamin B6 exhibit a similar bioavailability independent of their functional groups,^28^ for PNG, an impaired bioavailability (50–58%) has been reported in different studies conducted in vivo and in vitro*.* ^23,25,29,30^ In a mixed diet, the bioavailability of 2.3 mg B6 consumed per day by American men was estimated at 75% via plasma PLP concentrations and 24 h urinary excretion^31,32^ and is lower for a plant-based diet due to a higher proportion of PNG. Previous observations regarding the vitamin B6 status of vegetarians and vegans have been inconclusive. Reported findings range from a 30% prevalence of B6 deficiency regardless of diet type,^33^ to a 16% deficiency rate specifically among vegans,^34^ significantly lower plasma PLP concentrations in vegetarians, and, in contrast, no significant differences compared to omnivorous control groups.^35^ A study conducted from 2005 to 2006 in the US found that 11% of the participants in the general population were deficient in B6, with high demographic variations.^36^ In recent European studies, 20% of the adolescent subjects showed insufficient PLP plasma levels (≤30 nmol/L), while 5% were B6 deficient (≤20 nmol/L).^37^

Despite the findings from various bioavailability studies, so far, only a few methods have enabled the separate quantification of PNG or other B6 glycosides in their native form due to the unavailability of analytical standards.^38^ Most studies implementing LC–MS/MS quantification can be found in multivitamin analysis, where, e.g., all water-soluble vitamins were measured simultaneously, however, without including all important vitamers of each vitamin group.^39–43^ When the B6 glycosides are of interest, enzymatic treatment with taka-diastase or β-glucosidase is necessary, which neither allows insight into the type of sugar bound to B6 nor into the position of the glycosylated hydroxyl group.^44,45^ Additionally, gentle sample preparation techniques are required in order to leave the glycosidic bond intact, excluding currently prevalent methods using 1.0 M HCl and autoclaving at 120 °C. Conventional HPLC methods determine the total B6 as PN after dephosphorylation by acid phosphatase and conversion of PL and PM into PN.^46^ For the codetermination of B6 glycosides, β-glucosidase treatment and quantification as PN, PL, and PM after acid hydrolysis and dephosphorylation is suggested.^47^

Facilitated by modern *Koenigs-Knorr* reaction routes, the synthesis of different B6 glycosides provided the necessary standard compounds, including ^13^C_6_-PNG as an internal standard and various saccharides such as maltose linked to PN via a 5′-β-glycosidic bond.^48,49^ For the analytical gold standard method, i.e., stable isotope dilution analysis (SIDA) with LC–MS/MS detection, the addition of isotopically labeled standards improves the recovery and compensates for workup losses or measurement errors.^50^ We previously reported a SIDA method for the quantification of PN, PM, PL, PMP, and PNG in plant-based food.^38^ However, for the incorporation of further B6 glycosides, such as PN-5′-β-maltoside (PN-Malt) and PN-5′-β-cellobioside (PN-Cell), the chromatographic method necessitated improvement regarding analyte separation and retention. The structures of the analytes included here are depicted in Figure 1.

**Figure 1:**
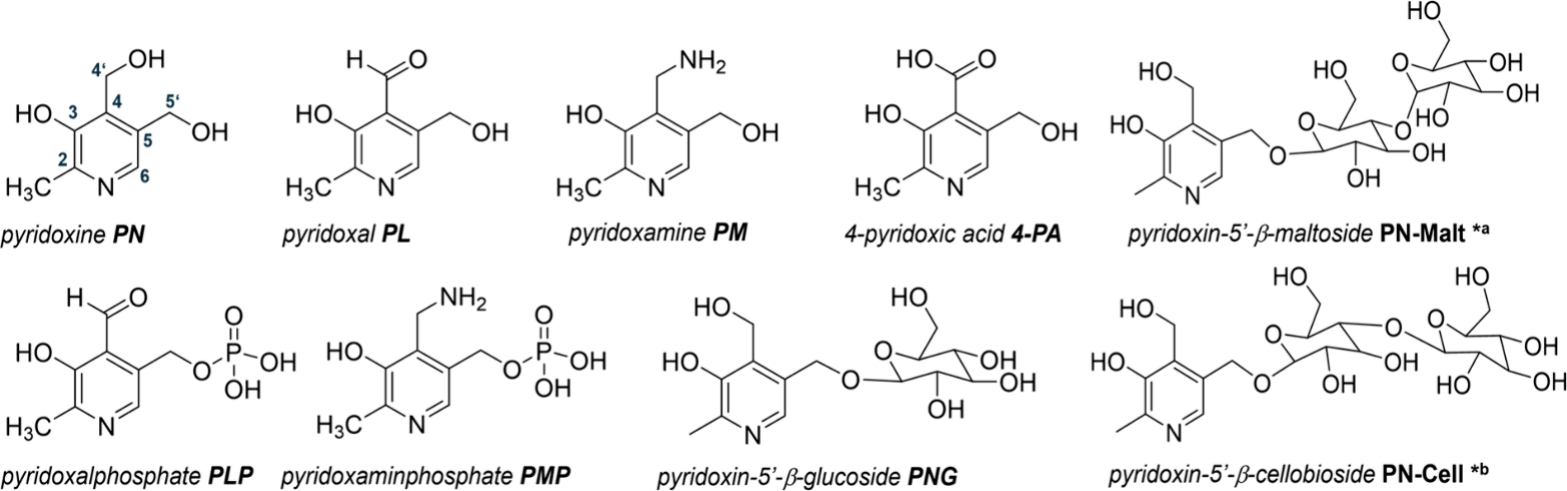
Structural formulas of the vitamers are relevant to this study. The vitamin B6 group comprises substrates sharing a common 2-methyl-3-hydroxypyridine scaffold with varying functional groups at C4′ and phosphorylation or glycosylation at C5′.

Barley, wheat, and brewer’s yeast, common brewing ingredients in Germany, have been reported as relevant sources of vitamin B6.^51–53^ While B-vitamins in beer in general originate from barley, their concentration can increase during germination and remains stable despite high temperatures in the kiln.^54,55^ For the B6 content in beer, varying data have been published, ranging from low amounts (15–375 μg/L^56^; 89.5–138.7 μg/L^57^) to higher concentrations (499–697 μg/L) with minor differences between beer types.^58^ Regarding the influence of technological steps such as alcohol removal and filtration, no studies have been reported on vitamin B6. In light of the aforementioned health-motivated increase of alcohol-free alternatives in the craft beer market, an in-depth understanding of the behavior of various high-value nutrients harbors the opportunity to market and advertise such products.

For all the reasons mentioned above, the aim of the study presented here was to develop and validate a stable isotope dilution assay for B6 vitamers, including various B6 glycosides, and apply it to a representative set of alcohol-free and full-strength beers from Germany.

## 2 Materials and Methods

### 2.1 Chemicals, Standards, and Solutions

The aqueous LC–MS buffer (5 mmol/L NH_4_ formate+0.2% FA) for mobile phase A was prepared by diluting a NH_4_ formate stock solution (10 M, LC–MS grd ≥ 99.0%, Sigma Aldrich, USA) with water (LC–MS grd., Th. Geyer, Germany) under the addition of formic acid (FA; LC–MS grd. ≥ 99.0%, VWR Chemicals, USA). Mobile phase B was prepared by adding 0.2% of FA to MeOH (LC–MS-grd≥ 99.9%, Honeywell, USA). All solvents used for LC–MS were sonicated before use. For sample preparation, the extraction buffer consisted of an aqueous phase (50 mmol/L NH_4_Formate + 5% FA) and ACN (LC–MS-grd ≥ 99.9%, Honeywell, USA) (40/60; v/v).

As analytical standards, the following substances were used: PN × HCl (99.0%, Alfa Aesar, USA), PL × HCl (>99.5%, Sigma Aldrich, USA), PM × 2HCl (≥98.0%, Sigma Aldrich, USA), PLP × H_2_O (≥97.0%, Sigma Aldrich, USA), PMP (≥98.0%, Sigma Aldrich, USA). The synthesis of 4-pyridoxic acid (4-PA) is described in the supplementary information (chapter 1). All remaining standards (PNG, PN-Malt, PN-Cell, ^13^C_6_-PNG, ^13^C_3_-PL, and ^13^C_3_-PN) were synthesized as previously reported.^48,49,59^

### 2.2 General Experimental Information

All experiments were carried out under subdued light to prevent analyte degradation. For exact determination of standard concentrations, analytical and internal standards underwent quantitative ^1^H NMR analysis (Bruker Advance II 400 MHz) in D_2_O (99.9 at. % D, Sigma-Aldrich, USA) in NMR tubes (177.8 × 4.97 mm, Bruker, USA). A 0.5 mmol/L L-tyrosine solution (>99.0%, Fluka Analytical/Sigma Aldrich, Germany) was prepared in 5 mL D_2_O and measured as a standard for the calibration of the NMR software (Topspin, Bruker). In addition, all standards were checked for cross-contamination by LC–MS/MS beforehand. Stock solutions were prepared for each analyte individually at 1 mg/mL, dried under nitrogen at 40 °C, and stored at −20 °C until application. Working solutions in concentration ranges between 0.01 and 100 μg/mL were kept in H_2_O/0.1% FA at −20 °C. These conditions were continuously monitored for analyte stability. Every centrifugation step was performed in a 5810 R centrifuge (Eppendorf SE, Germany) at 4 °C and 3220 g. Before LC–MS injection, each sample underwent membrane filtration (PVDF-20/13 filter, Ø 13 mm pore-size 0.2 μm, Macherey-Nagel GmbH, Germany). Equilibration of the sample, extraction buffer, and internal standards was achieved by vortex treatment (Vortex-Genie 2, Bender & Holbein AG, Switzerland).

### 2.3 Optimization of Sample Preparation

Beer samples (*n* = 69) were obtained from local supermarkets. Alcohol-free beers are defined as a group of beer containing ≤0.5 % vol ethanol. Of these, two subgroups were identified according to the beer labels as (i) alcohol-free lager with low (587.3 ± 124.4 μg/L) and (ii) high (339.2 ± 42.1 μg/L) residual sugar, respectively. Approx. 100 mL were degassed by ultrasonication for 30 min (VWR Intern., Radner, PA, USA), and stored in the absence of light at −20 °C until use. As a basis for the workup procedure, the methods by Bachmann et al. and Romanini et al.^38,57^ as constituents of the beer matrix would heavily contaminate the LC–MS/MS ESI-source, the amount of interfering substances must be minimized beforehand. Therefore, three beer types (lager, wheat beer, and dark bock beer) were chosen to represent the sample pool. For matrix reduction, different compositions of extraction buffer, varying dilutions, and pretreatment of the beer with organic solvents, as proposed by Scheibenzuber et al., were tested.^60^ Optical PDA detection at 190 nm allowed visualization of the unspecific absorption of matrix compounds during the LC runs. At the beginning of every sample workup for optimization, internal standards (final concentrations: ^13^C_6_-PNG, ^13^C_3_-PL: 20 μg/L; ^13^C_3_-PN: 50 μg/L) were added to the beer sample and thoroughly vortexed.

#### 2.3.1 Variation 1: “Dilute and Shoot 1:5”

To 1.0 mL of a beer sample spiked with internal standards, 4.0 mL of a solution consisting of 95% mobile phase A and 5% ACN (v/v) was added and thoroughly vortexed. Afterward, the solution was centrifuged for 15 min, and 1 mL of the ensuing supernatant was removed and membrane-filtrated before LC–MS/MS injection.

#### 2.3.2 Variation 2: “Dilute and Shoot 1:5”

A 0.5 mL beer sample with internal standards was mixed with a 2 mL solution of 40% mobile phase A and 60% ACN (v/v). After centrifugation, the supernatant was transferred into a 4 mL amber vial and dried under a N_2_-stream at 40 °C. The residue was reconstituted in 2.5 mL of mobile phase A, membrane-filtered, and injected into the LC–MS/MS.

#### 2.3.3 Variation 3: “Dilute and Shoot 1:10”

A similar approach to variation 1 with the use of 9 mL of extraction buffer instead of 4 mL was applied.

#### 2.3.4 Variation 4”: Dilute and Shoot 1:10”

A sample of 0.4 mL of beer spiked with IS was mixed with 3.6 mL of 40% mobile phase A and 60% ACN. After 15 min centrifugation, the supernatant was transferred into a 4 mL amber vial and dried under a N_2_-stream at 40 °C. The residue was reconstituted in 4.0 mL mobile phase A, and 1 mL of the solution was membrane-filtered and injected for LC–MS/MS measurement.

#### 2.3.5 Variation 5: “Dilute and Shoot 1:5 + Organic Extraction with Organic Solvents”

a) Cyclohexane b) Diethyl ether

In a 15 mL centrifuge tube, 1.0 mL of beer, IS, and 1 mL of organic solvent were homogenized by vortexing for 5 min. The emulsion was separated by 15 min of centrifugation and left at 4 °C overnight to enhance phase separation. After partition, the organic phase was dried under a N_2_ stream at 40 °C, reconstituted in 1 mL of mobile phase A, membrane-filtered, and measured by LC–MS/MS to monitor the carry-over of analytes. The workup procedure from there was identical to variation 1 for the aqueous phase.

#### 2.3.6 Variation 6: “Dilute and Shoot 1:5 + Organic Extraction with Organic Solvents”

a) cHex b) Diethylether

In a 15 mL centrifuge tube, 1.0 mL of beer sample, IS, and 1 mL of organic solvent were homogenized by vortexing for 5 min. The emulsion was separated by 15 min centrifugation and left at 4 °C overnight to enhance phase separation. Thereafter, the organic phase was discarded. In the aqueous phase, 0.5 mL was transferred into a new centrifuge tube, and the workup proceeded according to variation 2.

#### 2.3.7 Variation 7: Conventional B6 Sample Preparation^38^

1.0 mL beer sample was mixed by vortexing with IS and extraction buffer and centrifuged for 15 min. The supernatant was transferred to a 4 mL amber vial and dried under a N_2_ stream at 40 °C. The residue was reconstituted in 1 mL MS-buffer, membrane-filtered, and measured via LC–MS/MS.

#### 2.3.8 Variation 8: Conventional B6 Sample Preparation with Organic Extraction

a) Cyclohexane b) Diethyl ether

The procedure was identical to variation 5 with ensuing workup according to variation 7.

### 2.4 Final Sample Preparation

The standard sample volume was set to 400 μL, except for dark and bock beer, for which 200 μL was used. The samples were analyzed in triplicate. Internal standard concentrations varied between 20 and 100 μg/L, depending on the beer type and analyte. Special attention was paid to the linear range of the calibration graphs for the internal standard spiking levels.

After adding 1.0 mL of extraction buffer, the solution was thoroughly homogenized by vortexing before 2.0 mL of ACN was added. The precipitate was separated by 15 min centrifugation, and the supernatant was transferred into an amber vial, where it was dried under N_2_ stream at 40 °C. The samples were kept dry at −20 °C in amber vials for storage. Before the measurement, the residue was reconstituted in 1 mL of mobile phase A and membrane-filtered.

### 2.5 Instrumental Analysis

The analytical measurements were performed on a triple quadrupole mass spectrometer (LCMS-8050, Shimadzu, Kyoto, Japan) operated in the ESI-positive mode with scheduled multiple reaction monitoring (MRM) for all compounds. For quality control and compound identification, retention time (Rt) and the quantifier/qualifier ratio of the MRM transitions were frequently checked for all analytes in the samples and compared with the corresponding standard compound. Across all runs, the analytes met the ± 30% acceptance criterion for ion-ratio reproducibility, as summarized in supplementary information S23. Before this study, optimization of the ion source parameters for all analytes met the following conditions: Interface temperature 395 °C, desolvation line temperature 125 °C, heating gas flow 12.0 L/min, drying gas flow 8.0 L/min, nebulizing gas flow 3.0 L/min, interface voltage 4.0 kV. The optimized, analyte-specific conditions, collision energy, voltage, retention time (Rt), and MRM (multiple reaction monitoring) transitions can be found in supplementary information S13. The column oven temperature was set to 40 °C. Simultaneously, optical detection via a photodiode array (PDA; Nexera N2 SPD-M30A, Shimadzu, Kyoto, Japan) was executed. Chromatographic separation of the analytes was achieved on an ACQUITY Premier HSS-T3 column (2.1 × 100 mm, 1.8 μm; Waters, Milford, USA) with a matching ACQUITY Premier HSS-T3 precolumn (2.1 × 5 mm, 1.8 μm; Waters, Milford, USA) using the Nexera X2 UHPLC system (LC-30AD; Shimadzu, Kyoto, Japan) at a 0.4 mL/min flow rate. The binary LC-gradient started at 1.0% mobile phase B until 2.5 min, increasing to 2.0% B in 0.2 min, and the composition remained until 4.0 min. Subsequently, the solvent B concentration increased to 4% in 0.2 min, remaining constant until 7.5 min. From there, an increase to 99% phase B was achieved in 2 min, where it remained for 11.5 min. In 2 min, solvent B was decreased to the 1% starting conditions, where it stayed for 4.5 min until the end of the run at 18.0 min for equilibration. The autosampler (Nexera X2 SILC-30AC; Shimadzu, Kyoto, Japan) was constantly cooled to 4 °C. Improved peak shape was achieved by coinjection of 20 μL of mobile phase A before and after a sample injection volume of 10 μL. By the implementation of an additional valve between PDA and LC–MS, the introduction of the solvent flow into the mass spectrometer was set to 0.9–7.5 min.

### 2.6 Method Validation and Statistical Analysis

#### 2.6.1 Blank Matrix

Recovery and limit of detection (LOD) were determined by spiking a blank matrix with different analyte concentrations. Additionally, the calibration graphs of analytes without an IS identical in structure (PM, PN-Malt, PN-Cell, 4-PA) had to be matrix-matched to compensate for workup losses or matrix effects during measurement. Therefore, a blank matrix with properties similar to beer was developed, consisting of D­(+)-glucose (384.2 mg; anhydrous for biochemistry; Merck, Germany), maltose-monohydrate (357.5 mg; ≥95%, Merck, Darmstadt, Germany), and dextrin (4.003 g; dextrose eq 8–15 maltodextrine, Sigma-Aldrich, St. Louis, USA) in 200 mL of deionized water. The color was set to EBC 7 with sugarculeur to include Maillard reaction products. For the preparation of sugarculeur, 100 g of D­(+)-sucrose (≥99% analytical grade, AppliChem, Darmstadt, Germany) were caramelized in a pot for approximately 5 min until a dark brown color was reached. Then, 50 mL of deionized water was added, and the syrup was left to thicken. The surrogate blank matrix was checked for interfering peaks at the MRM-transitions of the analytes (see suppl. information S12).

#### 2.6.2 Calibration

For the calibration of the LC–MS/MS method, 12–17 calibration points with constant amounts of IS and varying quantities of analyte (A) were measured as a response curve. The compounds with an IS of identical structure (PN, PNG, and PL) were diluted in mobile phase A. Matrix-matched calibration was conducted for the remaining analytes (PM, PN-Malt, PN-Cell, and 4-PA). Before sample cleanup, the blank matrix was spiked with varying amounts of A and constant quantities of IS. The molar ratios *n*(A)/*n*(IS) were between 0.01 and 50.0, depending on the analyte (see suppl. information S14). Every mixture was injected in triplicate, and the average value was used for calculations. By plotting the molar ratios n­(A)/n­(IS) against the peak area ratios A­(A)/A­(IS), we performed linear regression analysis. Mandel’s test was conducted to check linearity and linear range.^61^

#### 2.6.3 Limit of Detection (LOD) and Limit of Quantification (LOQ)

Based on the specifications of DIN 32645,^62^ preliminary LODs and LOQs were estimated from the calibration graphs. Subsequently, LODs and LOQs were determined according to Vogelgesang and Hädrich,^63^ with the lowest addition level close to the LOD estimated as three times the signal-to-noise ratio. The blank matrix was spiked at four different concentration levels with unlabeled analytes PN (1.00, 4.00, 7.00, and 10.0 μg/L), PNG/PN-Malt/PN-Cell (0.20, 0.80, 1.40, and 2.00 μg/L), PL (10.0, 40.0, 70.0, and 100 μg/L), and 4-PA (0.75, 3.00, 5.25, and 7.00 μg/L). The highest addition level amounted to ten times the lowest concentration, which was slightly above the estimated LOD, and the remaining two levels were evenly distributed in between to obtain a balanced recovery function. As described above, the isotope-labeled standards were added at the following concentrations before sample cleanup: ^13^C_3_-PN 10.0 μg/L, ^13^C_3_-PL 20.0 μg/L, ^13^C_6_-PNG 2.00 μg/L. Every spiking level was prepared in triplicate.

#### 2.6.4 Recovery

In addition to the recoveries calculated for the LODs, the blank matrix was spiked with four different analyte concentration levels, representing the expected values in the sample set. Different spiking levels were chosen for the analytes PN (10.0, 100, 500, and 1000 μg/L), PM (7.50, 20.0, 50.0, and 100 μg/L), PL (10.0, 40.0, 50.0, and 100 μg/L), PNG/PN-Malt/PN-Cell (2.00, 10.0, 100, and 200 μg/L), and 4-PA (7.00, 10.0, 50.0, and 100 μg/L). Before sample cleanup as described above, the IS were added at the following concentrations: ^13^C_3_-PN 10.0 μg/L, ^13^C_3_-PL 50. μg/L, ^13^C_6_-PNG 20.0 μg/L. Recoveries were calculated as the ratio of determined and added concentrations.

#### 2.6.5 Precision

Workup and injection repetitions were performed to estimate the method’s precision concerning intraday, interday, and interinjection precision. For a broader representation of the sample set, two different beers (lager and wheat beer) were selected. Intraday precision was determined from a workup in triplicate (*n* = 3) with triplicate injection (*i* = 9) in one day. The interday precision consisted of three independent triplicate extractions (*n* = 9) over three weeks with triplicate injection (*I* = 27). One sample (*n* = 1) was injected nine times in a row (*i* = 9) for interinjection (machine) precision.

#### 2.6.6 Data Processing

The developed LC–MS/MS method, including the workup procedure, was validated according to the specifications of.^63^ Integration and evaluation of all LC–MS/MS data were conducted using LabSolutions software version 5.91 (Shimadzu Europe GmbH). Statistical analyses (averages, standard deviations (SD), relative standard deviations (RSD)) were performed in Microsoft 365 Excel, while outlier test, Shapiro–Wilk test for normal distribution, Levene’s test for variance homogeneity, and ANOVA with Tukey’s HSD test were performed via R 4.3.2. All calibration graphs were checked for linearity using Mandel’s test. The analysis was performed in triplicate with a triplicate injection for method validation and sample preparation. All statistical tests were conducted at α = 0.05. Before applying a *t*-test to compare data sets, the values were checked for variance homogeneity, outliers, and normal distribution. Accordingly, outliers were excluded from the statistics, and the *t*-test was performed to determine variance homogeneity or inhomogeneity. Total vitamin B6 content was calculated as a sum of the bioactive vitamers PN, PM, and PL, and the glycoside content was converted into PN equivalents by a molecular mass ratio. As a degradation product with no vitamin activity, 4-PA was not included.

## 3 Results and Discussion

### 3.1 Method Development

For the LC–MS method, a Waters HSS-T3 modified reversed-phase column was selected due to its improved affinity to polar analytes and references for retaining B6 in the literature.^56,57^ The optimal separation of the analytes, with particular attention given to the glycosides, was achieved with a 5 mmol/L NH_4_Formate buffer containing 0.2% formic acid. The optimized MRM transitions used as quantifier and qualifier ions are summarized in Table 1. Figures and tables portraying in-depth data of the method development are summarized in the Supporting Information (S6–S13).

**Table 1:** Precursor Ions and Product Ions of the Analytes and Internal Standards Used for Quantitation with Their Respective Optimized Retention Times (Rt)

analyte	Rt [min]	precursor *m*/*z*	product 1 *m*/*z* quantifier	product 2 *m*/*z* qualifier
PN	2.34	169.80	134.15	152.15
^13^C_3_-PN	2.33	172.80	137.15	155.15
PM	1.03	169.20	134.25	77.05
PL	1.76	168.20	150.20	94.15
^13^C_3_-PL	1.76	170.80	153.20	96.25
4-PA	4.43	183.75	148.15	166.20
PNG	3.44	331.90	108.20	152.20
^13^C_6_-PNG	3.04	337.90	108.20	152.20
PN-Malt	6.17	494.10	170.20	152.25
PN-Cell	6.42	494.10	170.25	152.15

In a preliminary experiment, a simple dilute and shoot method was tested for sample preparation, adapted from Bertuzzi et al. and Romanini et al.^56,57^ Due to the polarity of the analytes leading to weak retention for PM, diversion into the MS-waste at the beginning of the measurement was not possible, leading to heavy contamination of the ESI-source with matrix compounds. Hence, the workup procedure was optimized by lowering the dilution factor (1:5 for vars 1, 2, 5 and 6; 1:10 for vars 3 and 4) for the three beer types “lager”, “wheat beer”, and “bock beer” as a representation of the sample set. As expected, the signals were lower for the diluted approaches. However, the intensity decreased less than that expected from the dilution factor. Simultaneously, the matrix signal dropped approximately linearly with the dilution factor. This indicated a reduced matrix suppression despite lower analyte concentrations for the more diluted samples. Pre-extraction with organic solvents (cyclohexane var. 5, 6, 8 (a) and diethyl ether var. 5, 6, 8 (b)) for matrix reduction clearly showed a transition of analytes into the organic phase. In addition, the reduction of the matrix load in the aqueous phase was insufficient. Among the three beers used for method optimization, dark beer showed the highest matrix load. As a consequence of the preliminary experiments, the sample volume was reduced from 1 to 0.4 mL for all beers apart from strong and dark beers, where 0.2 mL was used instead. An additional extraction step with an organic solvent immiscible with water was dismissed due to analyte losses. As suggested by Bachmann et al., polar matrix compounds (e.g., proteins) were precipitated with 2 mL of ACN, which was mixed with the extraction buffer (1:2, extraction buffer: ACN, V/V) in the final method depicted in Figure 2. All diagrams describing the method optimization can be found in the supplementary information under chapter 2 (S6–S10).

**Figure 2:**
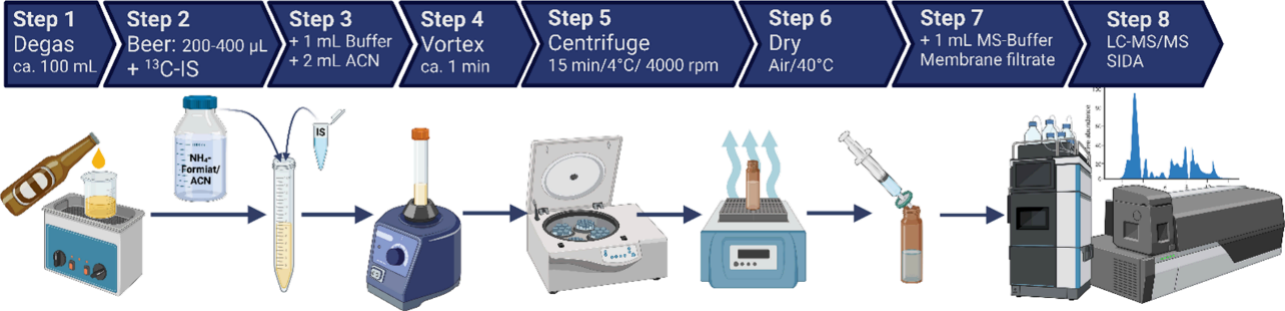
Schematic illustration of the beer sample preparation, Created in BioRender. Jahner, S. (2026) https://BioRender.com/066ep4q.

### 3.2 Method Validation

After injection of different mixtures of reference compounds and their respective internal standards, linear regression was performed by plotting n­(A)/n­(IS) on the *x*-axis against A­(A)/A­(IS) on the *y*-axis. Following the procedure by Vogelgesang and Hädrich,^63^ all equations were checked for their linear ranges, resulting in the linear equations, coefficients of determination, and calibration ranges documented in Table 2. The chromatogram of a wheat beer sample compared with the vitamin B6 standard mix is depicted in Figure 3. Extensive validation data are provided in the Supporting Information (S14–S23).

**Table 2:** Internal Standards, Regression Equation, Linear Range, *R* ^2^-Value (Coefficient of Determination) and LOD/LOQ for All Vitamin B6 Analytes

analyte	IS	regression equation	linear range *n*(A)/*n*(IS)	*R* ^2^	LOD [μg/L]	LOQ [μg/L]
PN	^13^C_3_-PN	*y* = 0.7626*x* – 0.0281	0.01–50.9	0.9996	0.27	1.06
PM^a,b^	^13^C_3_-PL	*y* = 1.0060*x* – 0.1358	0.02–24.9	0.9917	1.84	7.68
	^13^C_3_-PN	*y* = 0.1724*x* – 0.0257	0.01–10.2	0.9896		
PL	^13^C_3_-PL	*y* = 1.1794*x* + 0.0494	0.04–25.4	0.9976	2.94	12.9
4-PA^a^	^13^C_6_-PNG	*y* = 0.3988*x* – 0.0029	0.01–25.0	0.9996	1.26	4.87
PNG	^13^C_6_-PNG	*y* = 1.3190 + 0.0224	0.01–50.9	0.9993	0.07	0.31
PN-Malt^a^	^13^C_6_-PNG	*y* = 1.6560 – 0.0007	0.02–25.3	0.9999	0.08	0.31
PN-Cell^a^	^13^C_6_-PNG	*y* = 1.8259 + 0.0099	0.02–25.3	0.9999	0.05	0.20

**Figure 3:**
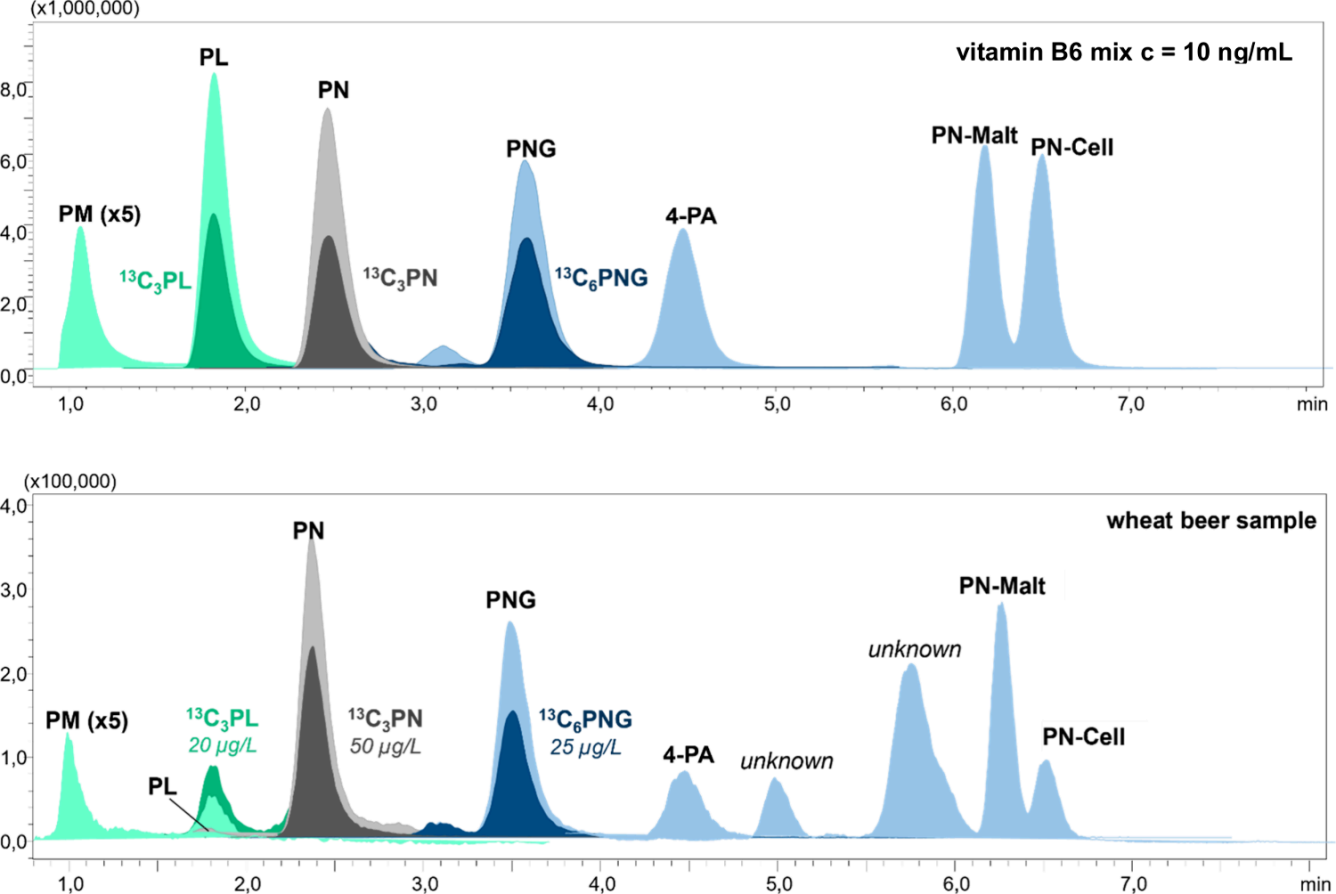
LC–MS/MS-chromatogram of a wheat beer sample (bottom) in comparison to a B6 standard mix (*c* = 10 ng/mL; top) including the internal standards ^13^C_3_-PL, ^13^C_3_-PN and ^13^C_6_-PN.

The LODs and LOQs determined for the method strongly depended on the type of analyte. While all B6 glycosides and PN showed LODs below 1.00 μg/L, the LODs of analytes with weaker retention, namely, PM and PL, were 1.84 and 2.94 μg/L, respectively. In the first two min, unspecific absorption could be observed in the UV trace at 190 nm, indicating matrix coelution, which explains the elevated LODs for these substances. For the oxidation product 4-PA, an LOD of 1.26 μg/L was determined. When determining the LODs, the RSD complied with the required limit of 10% for each spiking level and analyte (see suppl. information S21). Accordingly, the LOQs ranged between 0.20 and 1.06 μg/L for the B6 glycosides and PN and between 4.87 and 12.9 μg/L for 4-PA, PM, and PL. Comparable methods using LC–MS detection stated LODs of 0.63 μg/L for PN, PM, and PL in milk,^64^ 4.00 μg/L of PL in human milk,^41^ and 0.02–0.09 μg/L for 4-PA, PL, PM, and PN in human plasma.^65^ However, it must be considered that for the latter methods, the LODs were calculated as three times the signal-to-noise ratio (3x S/N), which generally results in more optimized values than our calibration line approach, as the latter also considers matrix effects and workup losses. A similar method on the same LC–MS/MS instrument quantifying fruits and vegetables yielded LODs between 2.80 and 18.0 μg/kg.^38^ So far, PNG has been commonly quantified as PN after enzymatic treatment. The only reported LOD determined by LC–MS/MS for PNG was stated as 10.0 μg/kg,^38^ moving in the same range as HPLC-FD (fluorescence detection), where the LOD for PNG was specified as 19.8 μg/kg for various food matrices.^24^ With the novel method presented here, the sensitivity for the glycosides was improved by 2 orders of magnitude due to the adjustment of the chromatographic retention and separation with the conditions tailored to B6 glycosides. So far, no LODs for PN-Cell and PN-Malt have been reported, but the values correlate well with those of PNG. Even though LOQs were relatively high for PL, PM, and 4-PA compared to the B6 glycosides, no impairment of the quantification was observed due to total B6 content ranging between 200 and 1100 μg/L in the beer samples. In addition, expanded uncertainties at the LOD and LOQ were estimated and are reported to provide a comprehensive assessment of the method performance (see Supporting Information S17).

In S18–S20, suppl. information chapter 3, the precision and recovery results are summarized. Compared to previous SIDA methods, including PNG,^38^ the precisions were improved, and RSDs fell below 5.1% for all analytes, thus fulfilling the required 10% limit^63^ and demonstrating the high accuracy of the method. Additionally, all recoveries, including concentration levels between LOD and LOQ for PL and PM, were between 70 – 120% as required.^63^ Apart from 4-PA with 85.8% and 82.7% recovery for two concentration levels, the recoveries ranged from 94 – 105% for the remaining analytes, confirming the ruggedness and reliability of the method. Although the assessment of robustness was not within the scope of this study, we expect the new method to be robust due to the inherent accuracy of the SIDA approach. However, this will be assessed in future work, e.g., by interlaboratory trials.

### 3.3 Vitamin B6 Content and Vitamer Distribution of Different Beer Types

For the screening of various beers, samples of different beer types (*n* = 65) were prepared and measured. The screening involved lager (*n* = 10), alcohol-free lager (*n* = 9), “Keller beer” (unfiltered lager; *n* = 7), wheat beer (*n* = 11), alcohol-free wheat beer (*n* = 7), pilsener beer (*n* = 5), bock beer (*n* = 8), dark beer (*n* = 5) and rice beer (*n* = 3). The average B6 content of every beer type was calculated as a sum of all vitamers excluding the degradation product 4-PA, which has no vitamin activity, with B6 glycosides converted to PN-equivalents. The contents are listed in Table 3 and depicted in Figure 4.

**Figure 4:**
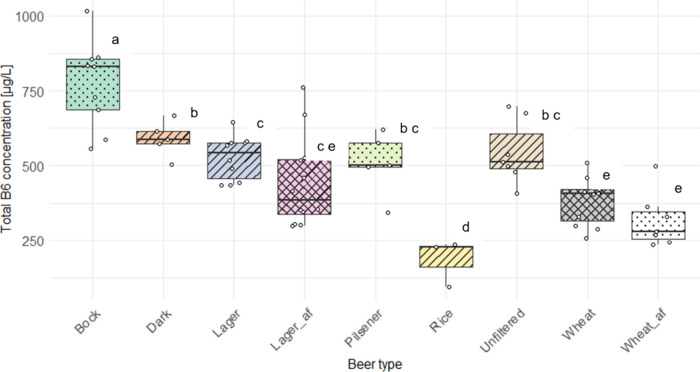
Distribution of average vitamin B6 concentrations across different beer types [μg/L]. The box plot displays the median, interquartile range (IQR), and potential outliers for each beer type, highlighting variation in the Vitamin B6 content, af alcohol-free.

**Table 3:** Number of Samples for Every Beer Type and Average Total B6 Content of Different Beer Types^a^

beer type	number of samples *n*	Ø total B6 content ± SD [μg/L]	Stat. group
*bock beer*	8	808.2 ± 154.0	^a^
*dark lager*	5	601.8 ± 67.4	^b^
*lager*	10	515.0 ± 80.6	^c^
*lager, alcohol-free*	9	461.8 ± 169.7	^c e^
*pilsener*	5	507.1 ± 106.5	^b c^
*rice beer*	3	185.3 ± 78.1	^d^
*unfiltered (“Keller beer”)*	7	544.6 ± 106.2	^b c^
*wheat beer*	11	424.6 ± 71.1	^e^
*wheat beer, alcohol-free*	7	342.5 ± 98.2	^e^

a The total B6 content was determined as the sum of all vitamers calculated as PN-equivalents for glycosides and excluding the degradation product 4-PA. Groups not sharing a common letter differ significantly according to Tukey’s HSD test (ANOVA, *p* < 0.05).

As shown in Table 3, bock beer had the highest total vitamin B6 content, followed by lager-type beers, dark beer and pilsener. The high vitamin B6 content in bock beer can be explained by a higher original wort (>16 °P) generated by larger amounts of malt used for this type of beer. Pilsener beer is adjusted to a stem wort of approximately 11 °P, which corresponds to only 69% of the original gravity in bock beer.^58^ By comparing the total B6 concentration of Pilsener and Bock beer, the difference amounts to 63%, thus indicating a connection between the original wort and B6 content. The overall lowest B6 concentrations were determined in wheat and rice beer. By comparison of the reported average B6 content of the corresponding raw materials, the order of the beer types correlates with these values. Hereby, polished rice (150 μg/100 g) represents the grain lowest in B6, while wheat contains 269 μg/100 g and barley 560 μg/100 g.^52^ According to the German purity law, wheat beer brewed in Germany must contain ≥50% wheat malt, with an original gravity of ≥11 °P, and the use of top-fermenting yeast.^66^ The other beer types are commonly bottom-fermented, but data on the influence of the yeast strain on the B6 concentration are not available. Hence, only tentative assumptions can be made by correlating the total vitamin B6 content in beer with the concentration in the raw material, as previously described.^55,67^ Further studies regarding malt types, brewing process control, and yeast strains should be conducted in the future.

ANOVA of the data set provided in total five different groups within the beer types (see Table 3 for clustering). While the high B6 bock beer and the low B6 rice beer showed a significant difference to all other sample sets, no significant difference was determined between dark lager, pilsener, and unfiltered beer; lager and alcohol-free lager, as well as lager, unfiltered beer, and pilsener; alcohol-free lager, wheat beer, and alcohol-free wheat beer. However, the small sample size within the groups of rice beer (*n* = 3) and dark beer (*n* = 5) needs to be acknowledged, thus limiting statistical significance regarding these specific subgroups. Two preliminary technological conclusions can be drawn from these results: (1) no B6 is lost during alcohol removal, and (2) no B6 is lost due to filtration.

The values obtained in this study correlate well with recent data obtained in literature,^58^ where a total B6 content as the sum of PM and PN of 613 ± 98.0 μg/L (Pilsener), 499 ± 157 μg/L (Wheat beer), 697 ± 116 μg/L (Export beer) and 594 ± 119 μg/L (Black beer) was determined by HPLC-FD after enzymatic treatment of the beer samples with taka-diastase and β-glucosidase. The total B6 concentration was slightly higher compared to that in our data. This could be due to the indirect determination of other, yet unknown, B6 glycosides by the unspecific enzymes applied in the latter study.^58^ In other recent studies, lower total B6 concentrations were found: Bertuzzi et al. quantified PN by LC–MS/MS with values between 15 and 376 μg/L in different beer types,^56^ whereas Romanini et al. (2021) determined even lower B6 concentrations in small and large scale brewing with the final content in beer as low as 89.5–138.7 μg/L as a sum of PN and PM by LC–MS/MS.^57^ There, PL was included in the method but could not be detected in any beer sample. However, neither of these studies used any enzymes to take into account the contribution of the glycosides, which partly explains the lower concentrations.

The distributions of B6 vitamers in the different beer types are listed in Figure 5. As expected from the literature data, no B6 phosphates were detected in any of the beer samples. The transitions of PLP and PMP were initially added to the method for qualitative screening, but were not included in the final method and the validation. Neither phosphorylated vitamers were detected within the method sensitivity. For PNP, no commercial standard is available, and it was also reported as insignificant to the total vitamin B6 in foods.^44^ Compared to the B6 content including PNG determined in grains, PLP plays no role in the total B6, while PMP rarely contributes to more than 10% of the overall B6_._ ^38,67,68^ Additionally, stability tests of B6 vitamers in buffer solutions conducted at our institute (unpublished) showed notable degradation of PMP and PLP at room temperature under subdued light, which increased upon light exposure due to high photosensitivity as suggested previously.^69^ The experiments also revealed an increasing degradation at higher storage temperatures. Therefore, when considering the harsh temperatures during mashing and wort boiling in the brewing process, phosphate breakdown of B6 is to be expected. Finally, the phosphatase activity system of *Saccharomyces cerevisiae* is well-researched, and at least two membrane-bound extracellular phosphatases have been reported.^70,71^ Accordingly, any B6 phosphates remaining in the final wort will likely undergo rapid enzymatic dephosphorylation upon yeast fermentation. Taken together, all of the findings comprehensibly explain the absence of B6 phosphates in beer.

**Figure 5:**
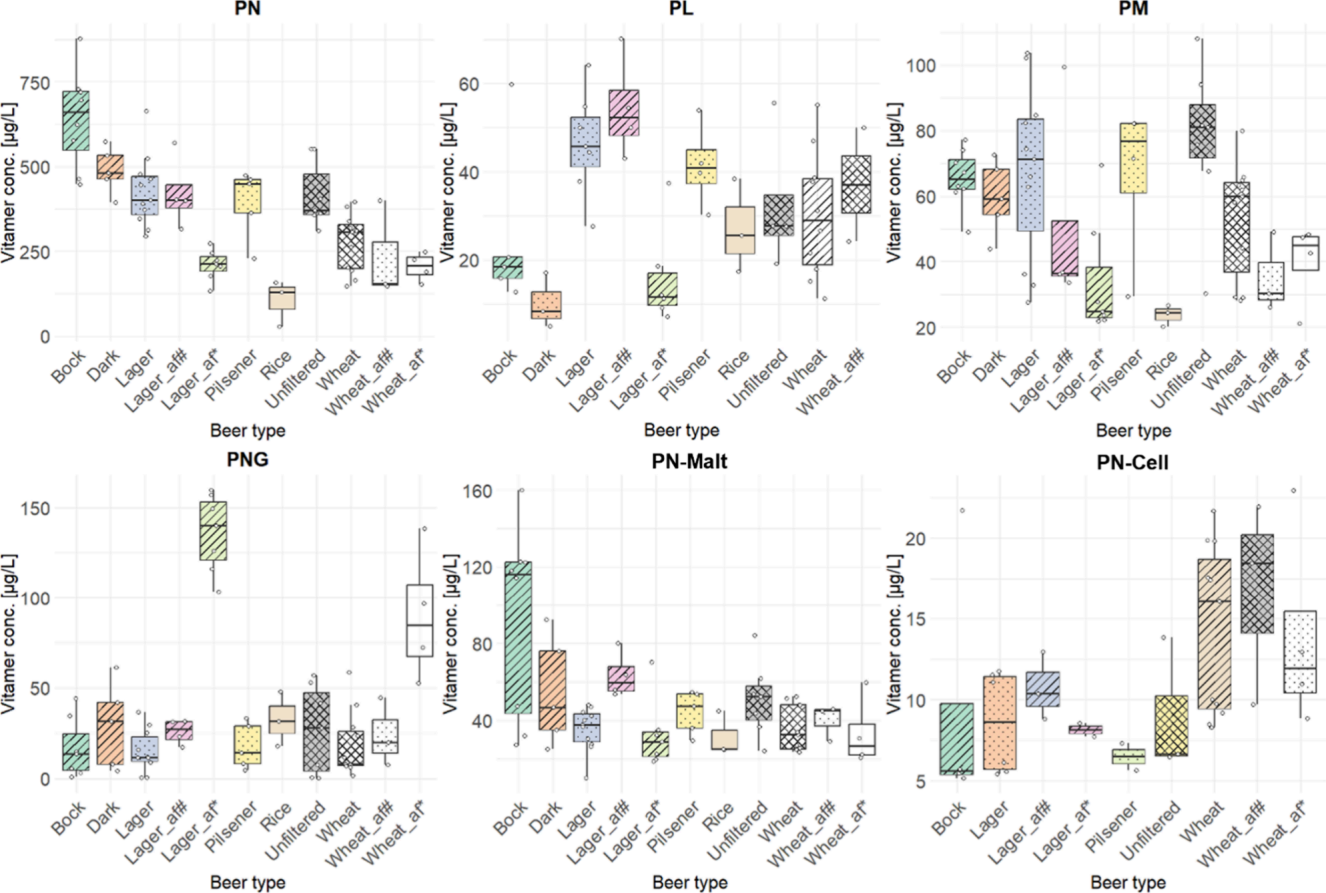
Distribution of B6 vitamer PN and PNG concentrations across different beer types [μg/L]. The box plot displays the median, interquartile range (IQR), and potential outliers for each beer type, highlighting variation in the vitamer content. For alcohol-free beer: # sugar content <2.0 g/100 mL; * sugar content ≥2.0 g/100 mL, af alcohol-free.

### 3.4 Alcohol-Free Beer

Even though, at first glance, the B6 content of alcohol-free lager appears lower compared to that of conventional lager, statistical testing revealed no significant difference between the two sample sets, presumably due to the more extensive scattering of the values for alcohol-free lager. Additionally, within the sample set of alcohol-free lager, two subgroups emerged as an exploratory observation: alcohol-free lager with low (587.3 ± 124.4 μg/L) and high (339.2 ± 42.1 μg/L) residual sugar as specified on the beer labels. In general, strategies for alcohol removal can be classified into two groups: biological and physical applications.^10,72^ Of these, the biological variant is likely to generate alcohol-free beer containing high residual sugar by restricted ethanol formation, applying arrested or limited fermentation.^73^ In recent years, approaches using specific yeast strains, which are unable to metabolize maltose (i.e., *Saccharomyces rouxii* *spp.*), have gained popularity to generate only minor amounts of ethanol from glucose, fructose, and sucrose.^74^ The latter represents another technology for alcohol-free beer with high sugar content. By contrast, low sugar levels in alcohol-free beer indicate the implementation of physical methods that follow complete fermentation. In a subsequent step, ethanol is removed by procedures such as vacuum distillation, membrane filtration, or reverse osmosis.^10^ In general, these methods are designed to gently remove alcohol and preserve substances sensitive to heat, such as aldehydes, which play an essential role in the aroma profile of beer. The data obtained in our experiments suggest no adverse effects of alcohol removal on the B6 concentration but rather indicate a positive impact of complete fermentation. For a more in-depth understanding, the vitamer distributions of the different beer types will be discussed in the following.

In Table 4, the native vitamer content is listed with PN being the main vitamer in every beer type. While PNG only holds a small share of the total B6 content for most beers, alcohol-free beer displays a substantially higher PNG concentration in mean. By comparing alcohol-free with regular lager beer, the PNG concentration is five times higher in the alcohol-free product. Focusing on alcohol-free beer, an additional correlation was observed: alcohol-free lager with a higher amount of residual sugar correlated with an increased PNG content (136.0 ± 21.5 μg/L) compared to alcohol-free lager with lower residual sugar (25.9 ± 7.0 μg/L). This suggests PNG degradation during the fermentation, presumably by yeast enzymes. The existence of membrane-bound and secreted extracellular β-glucosidase in various types of *Saccharomyces* has been debated, but the activity could also be attributed to a glucan-1,3-β-glucosidase.^75,76^ As the total vitamin B6 content of entirely fermented alcohol-free beer is significantly higher than in alcohol-free beer prepared by suppressed fermentation, the difference could either stem from additional glycosides such as putative PN-4′-β-glucoside^77^ or other B6 oligosaccharides^26,27^ that are potentially released during mashing or fermentation. Another option is the de novo biosynthesis of B6 in yeast metabolism. The latter, however, is the more unlikely explanation, as newly synthesized B6 would need to be released from the yeast cell into the beer. According to previous studies on the uptake of vitamin B6 in resting cells of *Saccharomyces carlsbergensis*, the intracellular concentrations can be up to 3000 times higher than those provided in the external medium due to an active proton symporter system of PN, PL, and PM.^78^ Additionally, metabolic accumulation of B6 vitamers by yeast cells was shown for 35 strains independently of their metabolic phase.^79^ In summary, these reports indicate little to no secretion of vitamin B6 in the medium.

**Table 4:** Native Mean B6 Vitamer Concentrations of Different Beer Types in [μg/L] ± SD; Alcohol-Free AF^a^

vitamer	bock beer	dark beer	lager	lager, AF	pilsener	rice beer	unfilt. beer	wheat beer	wheat beer, AF
PN [μg/L]	**642.8** ^a^ ± 144.6	**489.5** ^b^ ± 72.8	**403.2** ^b^ ± 77.4	**330.1** ^bc^ ± 150.6	**395.7** ^b^ ± 102.8	**105.6** ^d^ ± 67.9	**416.3** ^a^ ± 94.8	**264.9** ^e^ ± 81.3	**246.7** ^ce^ ± 95.7
PNG [μg/L]	**16.7** ^a^ ± 16.6	**29.4** ^b^ ± 22.0	**15.2** ^c^ ± 12.2	**68.6** ^cd^ ± 56.7	**17.8** ^cc^ ± 12.6	**32.6** ^f^ ± 15.1	**26.9** ^cb^ ± 23.1	**17.8** ^c^ ± 16.1	**43.1** ^dc^ ± 32.2
PN-Malt [μg/L]	**93.1** ^a^ ± 50.0	**55.0** ^b^ ± 26.2	**35.9** ^b^ ± 9.4	**44.7** ^b^ ± 19.7	**44.3** ^b^ ± 11.0	**31.6** ^b^ ± 11.5	**51.0** ^b^ ± 18.3	**36.5** **^b^** ± 11.8	**40.5** **b** ± 13.3
PN-Cell [μg/L]	**9.5** ^a^ ± 8.1	3.6* ± 0.04	**9.5** ^a^ ± 3.0	**10.3** ^a^ ± 1.8	6.5* ± 1.2	12.7* ± 1.0	**8.8** ^a^ ± 4.5	**14.9** ^b^ ± 5.2	**16.5** ^b^ ± 5.1
PM [μg/L]	**65.6** ^a^ ± 8.8	**59.6** ^ac^ ± 11.4	**70.8** ^a^ ± 24.7	**41.0** ^b^ ± 24.1	**66.3** ^a^ ± 25.1	**23.8** ^b^ ± 3.2	**76.9** ^a^ ± 24.6	**54.1** ^bc^ ± 17.8	**40.4** ^bc^ ± 7.8
PL [μg/L]	**25.5** ^a^ ± 19.4	**10.1** ^b^ ± 5.5	**50.6** ^a^ ± 10.5	**44.0** ^a^ ± 27.6	**41.5** ^a^ ± 9.8	**27.1** ^ab^ ± 10.6	**34.1** ^ab^ ± 16.7	**33.1** ^ab^ ± 15.5	**32.5** ^ab^ ± 13.2

a Horizontal groups not sharing a common letter differ significantly according to Tukey’s HSD test (ANOVA, *p* < 0.05). *: not included in ANOVA due to insufficient data.

### 3.5 Vitamin B6 Glycosides

As aforementioned, the well-characterized B6 glycoside PNG has been quantified in every beer type, with particularly high amounts in some alcohol-free beers. For the first time described in 1977,^80^ PNG is well documented as the most prevalent form of B6 in plants.^24,38^ In comparison to PN, bioavailability is compromised due to the glucose attached and has been found to be only 50–58%. The compound PN-5′-β-cellobioside was isolated from rice bran in 1988, but no further studies on the occurrence and abundance of the glycoside have been conducted.^26^ Here, it was detected in low concentrations in every beer type, ranging between 3.6 and 16.5 μg/L, with the highest content in wheat and rice beer. The presence in rice bran also explains its occurrence in rice beer. Due to the low PN mass percentage in PN-Cell (34% of the total molecular weight), its contribution to the total B6 content is marginal. No studies have been conducted to date regarding its bioavailability, but it can be assumed to be similar to or lower than that of PNG. By contrast, the concentration of the novel glycoside PN-Malt was notably higher in all beer types (31.6–93.1 μg/L) and most prevalent in bock beer. So far, the origin of the compound is inconclusive and can only be hypothesized. On the one hand, conjugation of PN to polysaccharides (e.g., starch) in the raw material is conceivable. Thereafter, the enzymatic liberation of PN-Malt during malt preparation (germination) or during mashing, where amylolytic enzymes are generated and activated, represents one possibility. As a result, α- and β-amylase break down α-1,4-bonds, creating maltose among other sugars and dextrins, to which PN can still be bound. On the other hand, de novo enzymatic synthesis of PN-Malt by thermally activated enzymes after the release of maltose in the mashing process is plausible.^73^ Why PN-Malt is more abundant than PNG in the final beer and not cleaved completely by yeast activity is unclear, as PNG is subject to a distinct breakdown compared to that of the raw materials. However, when comparing the molar ratios of both substances PNG and PN-Malt, the molar concentrations are almost similar for both glycosides (apart from bock beer), indicating either a molar concentration limit for yeast digestion, or higher affinity to maltose, which is present at higher concentrations (56–59% in the wort) and serves as a primary nutrient for the yeast.^73^ Another theory is that the formation of PN-glycosides is subject to an equilibrium reflecting the actual sugar content of the beer during its final processing steps. To further understand the origin and behavior of the glycosides, in-depth studies of the complete brewing process are necessary.

### 3.6 Contribution of Beer to the Daily B6 Uptake and Health Claim Declarations

According to the guidelines suggested by D-A-CH, the recommended daily intake (RDI) of vitamin B6 is 1.4 mg for women and 1.6 mg for men.^32^ In 2022, the per capita consumption of beer in Germany was estimated as 90 L per year, but calculated for persons above the legal drinking age of 16, the amount is supposedly higher. Due to the recent assumption that there is no threshold for safe ethanol consumption published by the WHO,^5^ the German Society for Nutrition advises a maximum ethanol intake of 10 g/day for women and 20 g/day for men, translating to approximately 250 mL of a 5 vol% Lager for women and 500 mL for men.^81^ Taking these considerations into account, an average Lager from our study per serving provides 16% of the RDI for men (258 μg/0.5 L) and 9.2% (129 μg/0.25 L) for women.

While the intake of alcohol-free beer is significantly lower, its consumption could contribute to the overall vitamin B6 uptake, as it is not subject to any limitation of alcohol intake. Using the average B6 content of alcohol-free lager with a low residual sugar content (587.3 ± 124.4 μg/L), one serving (0.5 L) contributes to 15% of the RDI for women and 13% for men. However, the alcohol-free lager with the highest B6 content (761.5 μg/L) already accounts for 27% of the RDI for women and 24% for men for the intake of one bottle (0.5 L). According to the European health claim regulation, 100 mL of the beverage needs to contain 7.5% of the RDI to allow for a claim of the B6 content on the label. This amounts to 1050 μg/L of alcohol-free beer for women and 1200 μg/L for men. Hence, a health claim would not yet be permitted for any of the beers we examined, as a declaration for alcoholic beverages is prohibited.^82,83^ Due to the German purity law, the world’s oldest food act, setting narrow boundaries for product innovation,^84^ technological considerations are paramount in achieving the concentrations necessary for health claims. While the sample set of rice beer was small in this study and needs to be considered with caution, the overall data obtained here proves evidently that beer brewed from barley has significantly higher overall B6 contents. Wheat beer, which is traditionally brewed with at least 50% wheat malt and the rest usually consisting of barley malt, also exhibited significantly lower B6 concentrations compared to Lager beer brewed with 100% barley malt.^66^ These data imply that beer brewed according to the German purity law (in which wheat beer is an exception) possesses a naturally higher overall B6 content, presumably depending on the raw material. Any influence of yeast type (top- or bottom-fermenting) still needs further investigation.

## Supplementary Material



## Notes

Chemical synthesis of 4-pyridoxic acid including ^1^H- and ^13^C NMR spectra, experimental results of the method optimization for sample preparation, extended validation data and LC–MS/MS settings, selected LC–MS/MS chromatograms, and a complete list of the B6 vitamer concentrations in each beer sample (PDF)
