# Supplementary material for: Quantitation of Vitamin B6 Vitamers and Glycosides in German Alcohol-Free and Full-Strength Beer by a Stable Isotope Dilution LC–MS/MS Method
Source: J Agric Food Chem. 2026 Apr 22;74(17):13924–35. doi: 10.1021/acs.jafc.5c14229 (PMC13154175; doi:10.1021/acs.jafc.5c14229)
Supplement: Supplementary file 1 [file jf5c14229_si_001.pdf]

Supporting Information

Quantitation of Vitamin B6 vitamers and glycosides in German alcohol-free and full-strength beer by a stable isotope dilution LC-MS/MS method

Simone Jahner[1], Elias Geilich[1], Carina Hagenauer[1] and Michael Rychlik[1,2] *

[1] Chair of Analytical Food Chemistry, Technical University of Munich, D-85365 Freising, Germany

[2] University of Queensland, Centre for Nutrition and Food Sciences, Brisbane, 4072, Australia

*** Correspondence:** Corresponding author: Michael Rychlik, michael.rychlik@tum.de

1. Chemical synthesis of 4-pyridoxic acid

Following the instructions published by (**1**), the three-step preparation of 4-pyridoxic acid (4-PA) from pyridoxine hydrochloride (PN*HCl) was optimized.

1.1 Oxidation of PN to PL and precipitation of PN-Oxime

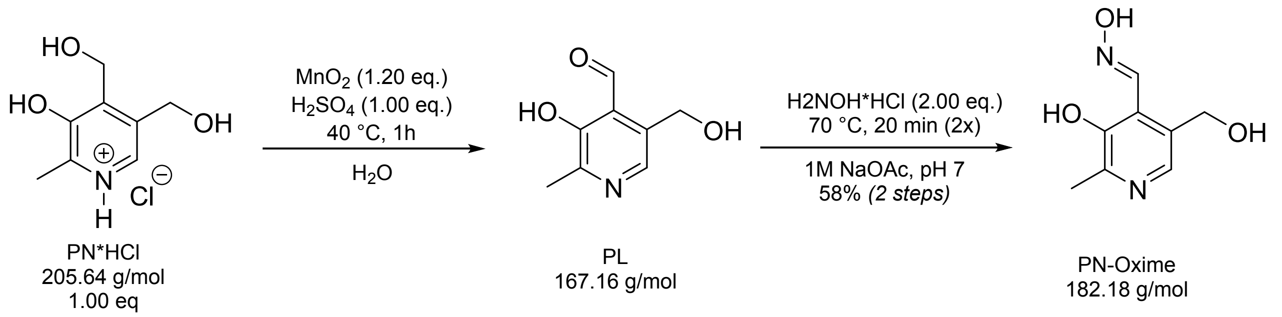

Figure S1. Reaction equation for the oxidation of PN to PL and subsequent precipitation of PN-Oxime.

PN*HCl (0.10 g, 0.49 mmol, 1.00 eq.; ≥ 98%, VWR Chemicals, Germany) was dissolved in 4 mL water (dest.). Then, 1.2 eq. MnO_2 (0.05 g, 0.58 mmol, 1.20 eq.; technical grd., Fluka, Germany) were suspended in the solution and 1.0 eq. H_2SO_4 (25.6 µL, 0.49 mmol, 1.00 eq.; 97%, VWR Chemicals, Germany) were added dropwise. The suspension was heated at 40 °C for 1 h until the MnO_2 was fully dissolved, with the end of the reaction marked by a color change from a brown suspension to a yellow solution. The solution was neutralized with 3M KOH (≥ 84.0%, Merck KGaA, Germany) before the precipitation of the PN-oxime.

By addition of a 1M sodium acetate buffer (5 mL, 10.0 eq.) to afford a stable pH 7 (NaOAc*3H_2O; Merck, Germany) and 2.0 eq. hydroxylamine hydrochloride (69.0 µg, 0.99 mmol, 2.00 eq.; H_2NOH*HCl; ≥ 98%, Fluka, Germany) the oxime was precipitated while heating to 70 °C for 20 min. Another 1 mL of the 1M NaOaAc-buffer (2.00 eq.) was added and heating at 70 °C for 20 min was repeated. For precipitation of the PN-oxime, the flask was put on ice for 10 min. The product was isolated by Büchner filtration, washed with cold water, and transferred into a round-bottom flask. In this vessel, the PN-oxime was dissolved in hot ethanol (absolute; 99.97%, VWR Chemicals, Germany), treated with activated charcoal (100 mesh particles; Sigma Aldrich, USA) for decolorization, and refluxed for approximately 5 minutes. The charcoal was then removed by Büchner filtration, and the ethanol was evaporated under reduced pressure using a rotary evaporator (BÜCHI Rotavapor R-200, BÜCHI Labortechnik AG, Switzerland). Yield: 58%

1.2 Oxidation of PN-oxime to PN-nitrile

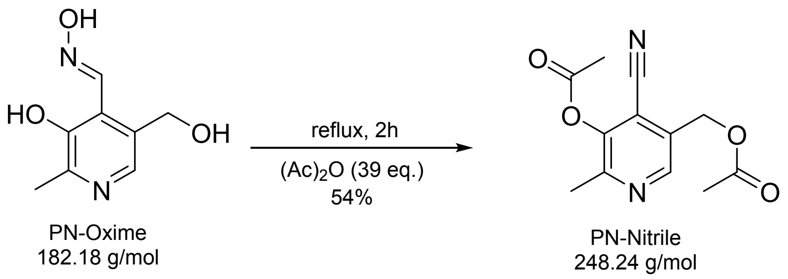

Figure S2. Reaction equation for the oxidation of PN-Oxime to PN-Nitrile.

The dried PN-oxime obtained in the previous step was dissolved in 0.95 mL acetic anhydride (39.0 eq.; ≥ 99%, Sigma Aldrich, USA) and refluxed at 140 °C for 2 hours. The resulting residue was dissolved in a small amount of ethanol under heating, and all volatile components were subsequently removed by rotary evaporation. The residue was extracted with diethyl ether and successively washed twice with saturated $NaHCO_3$ (≥ 98%, VWR Chemicals, Germany) solution, three times with water, and once with saturated NaCl (technical grd., VWR Chemicals, Germany) solution. The organic layer was dried over Na_2SO_4 (≥ 99%, Merck KGaA, Germany) and concentrated under reduced pressure. For recrystallization, the solid was dissolved in a mixture of ethanol and n-pentane (technical grd., VWR Chemicals, Germany) (1:1, v/v) and left to crystallize overnight in a freezer. The precipitated crystals were collected by Büchner filtration and washed with cold petroleum ether (technical grd., VWR Chemicals, Germany). For re-crystallization, the solid was dissolved in an EtOH/pentane mixture (1:1, v/v) and left to crystallize overnight in a freezer. The precipitated crystals of the PN-Nitrile (2-methyl-3-acetoxy-4-cyano-5-acetoxymethylpyridin) were collected by Büchner filtration and washed with cold petroleum ether. Yield: 54%.

1.3 Base-catalyzed hydrolysis of PN-nitrile to 4-PA

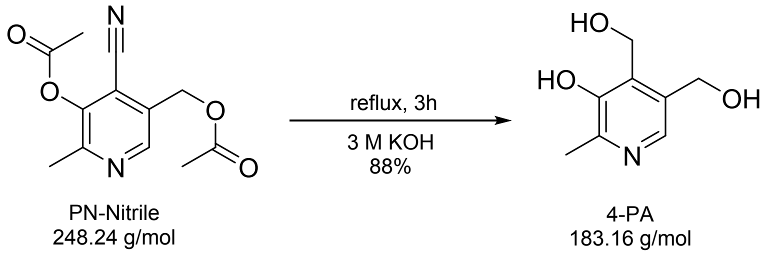

Figure S3. Reaction equation for the base-catalyzed hydroloysis of PN-nitrile to 4-PA.

The crystals obtained in step 2 were treated with 37 eq. of 3 M KOH and refluxed at 120 °C for 3 h. The reaction mixture was then neutralized on ice with 3 M HCl (32%; VWR Chemicals, Germany) until slightly acidic. The resulting 4-PA, which crystallized overnight in the refrigerator, was collected via centrifugation (5810 R centrifuge, Eppendorf SE, Germany at 4 °C and 3220 g) and detaching the supernatant. Consequently, the product was dried under vacuum on a rotary evaporator after removal of the corresponding solvent and subsequently subjected to high vacuum (≈ 0.2 mbar) via

Schlenk line for an additional 30 minutes prior to final weighing and yield determination. Yield: 88%; Total yield over all three steps: 25.1 µg (28%)

1.4 NMR analysis of 4-PA

For 1H-NMR analysis of the final product, 10.3 mg were dissolved in 800 µL of a 0.2% NaOD (40% in D2O; 99 atom% D, Sigma Aldrich, USA) solution in D_2O (99.9 atom% D, Sigma Aldrich USA), and 600 µL of this solution (7.7 µg) were measured in NMR tubes (177.8 × 4.97 mm, Bruker, USA). A 0.5 mM L-tyrosine (>99.0%, Fluka Analytical/Sigma Aldrich, Germany) solution was prepared in 5 mL D2O and measured as a standard for the calibration of the NMR software (Topspin, Bruker).

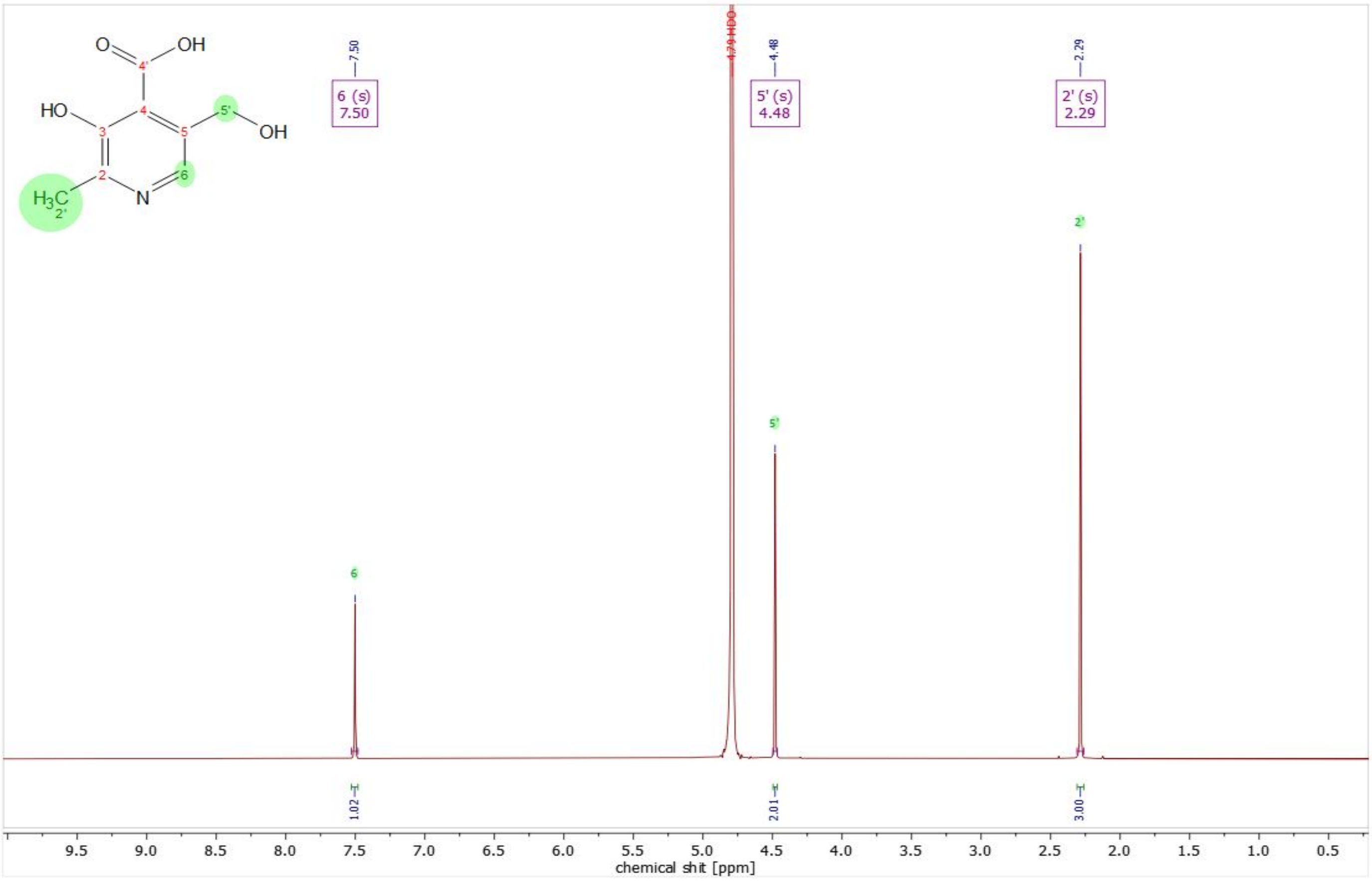

Figure S4. 1H-NMR (400 MHz, D2O/0.2% NaOD) of 4-pyridoxic acid.

^{1}H-NMR (400 MHz, D_2O/0.2% NaOD, 292 K): δ 7.50 (s, 1H, H6), 4.48 (s, 2H, 52‘), 2.20 (s, 3H, H2‘)

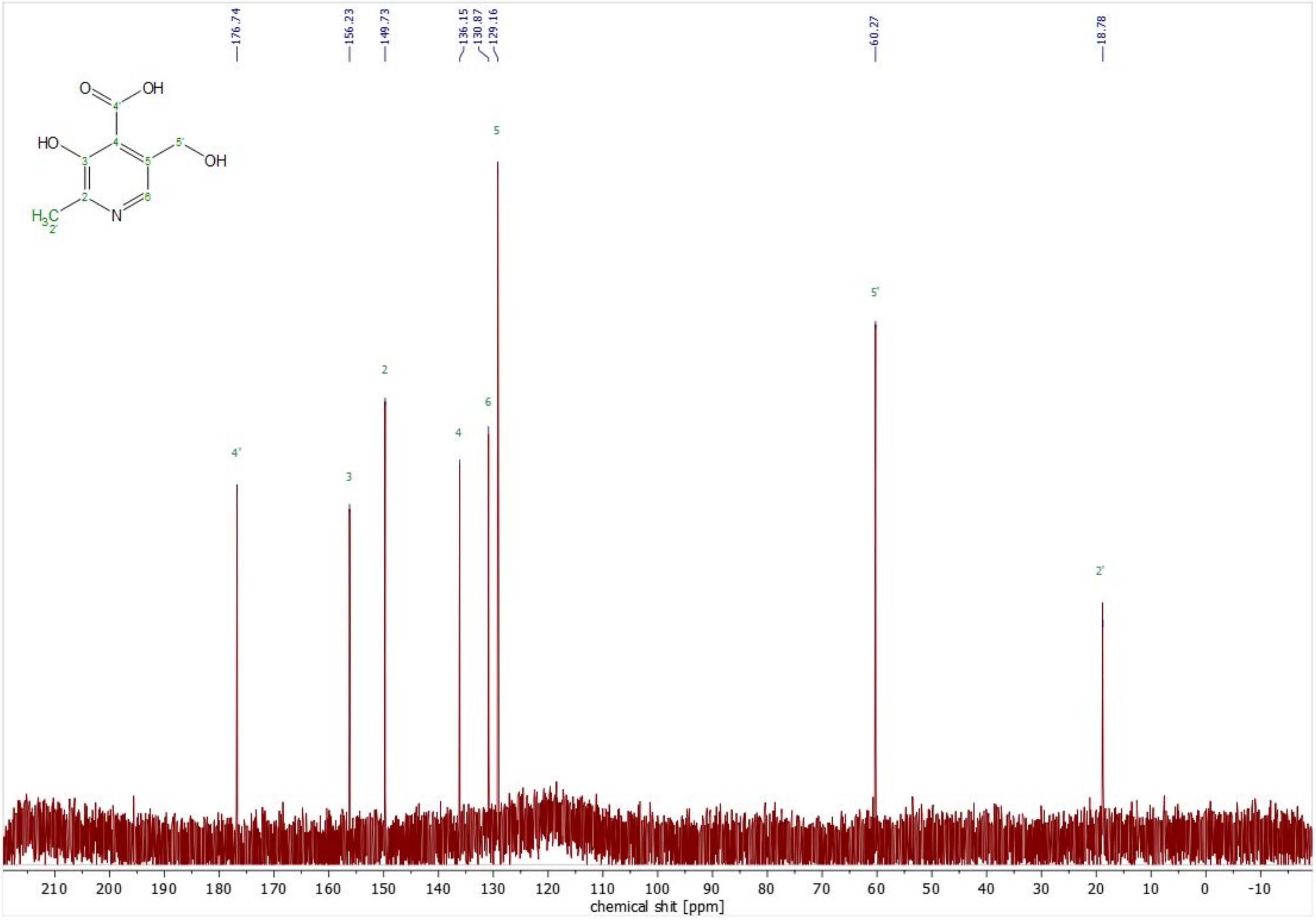

Figure S5. 13C-NMR (400 MHz, D_2O/0.2% NaOD) of 4-pyridoxic acid.

^{13}C-NMR (400 MHz, D_2O/0.2% NaOD, 292 K): 176.74 (C4’), 156.23 (C3), 149.73 (C2), 136.15 (C4), 130.87 (C6), 129.16 (C5), 60.27 (C5’), 18.78 (C2’).

ESI$^+$-MS calcd: 184.1 (+ H^+)

found: 183.8 (+ H^+)

2. Results of method optimization for sample preparation

2.1 Matrix Effects

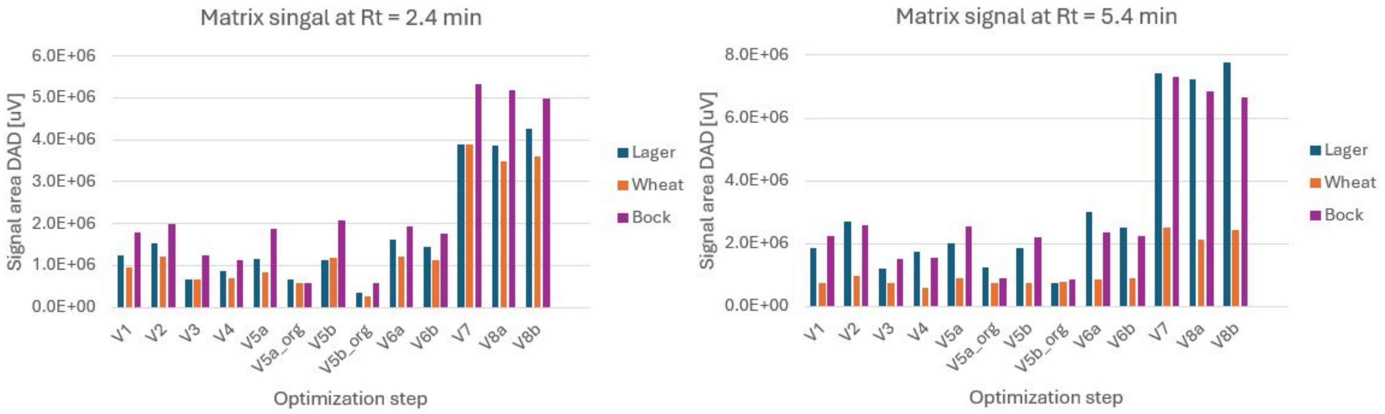

Figure S6: Comparison of the signal area [uV] of the two major matrix peaks at Rt=2.4 min and Rt=5.4 min for each optimization variation.

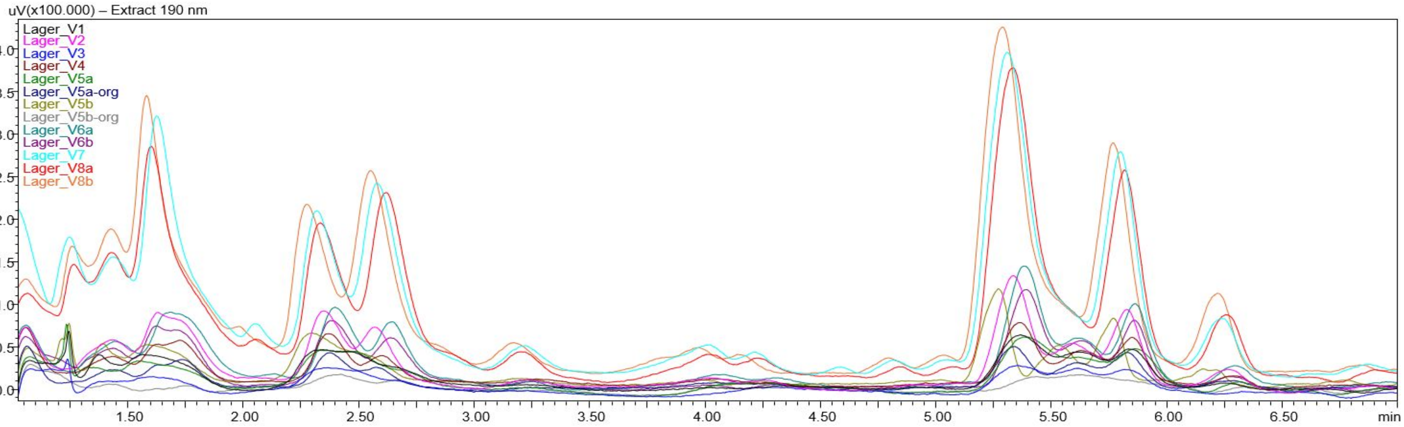

Figure S7. Extract of DAD UV at 190 nm for different sample preparation variations (lager beer).

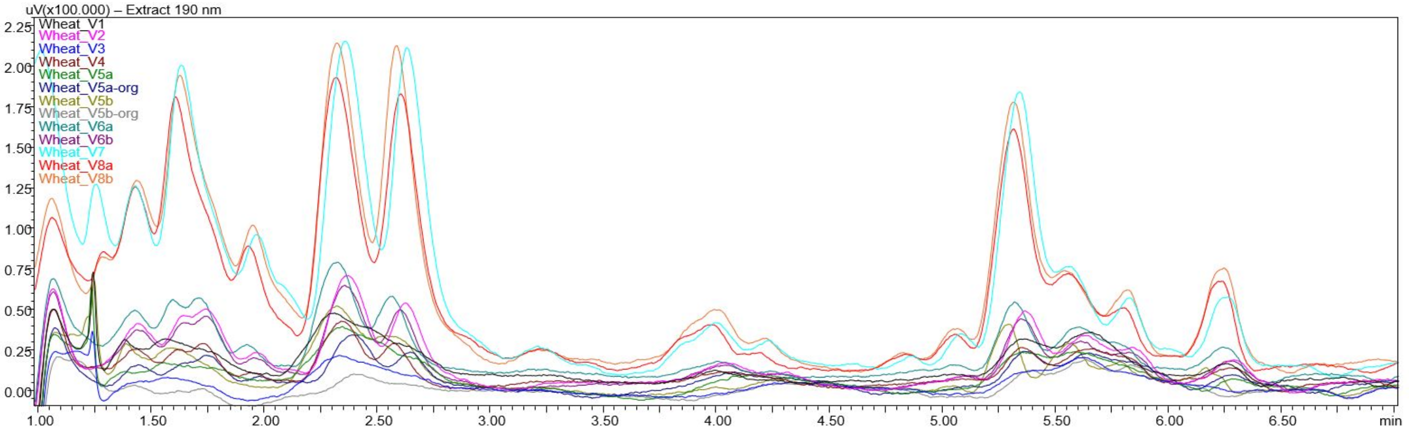

Figure S8. Extract of DAD UV at 190 nm for different sample preparation variations (wheat beer).

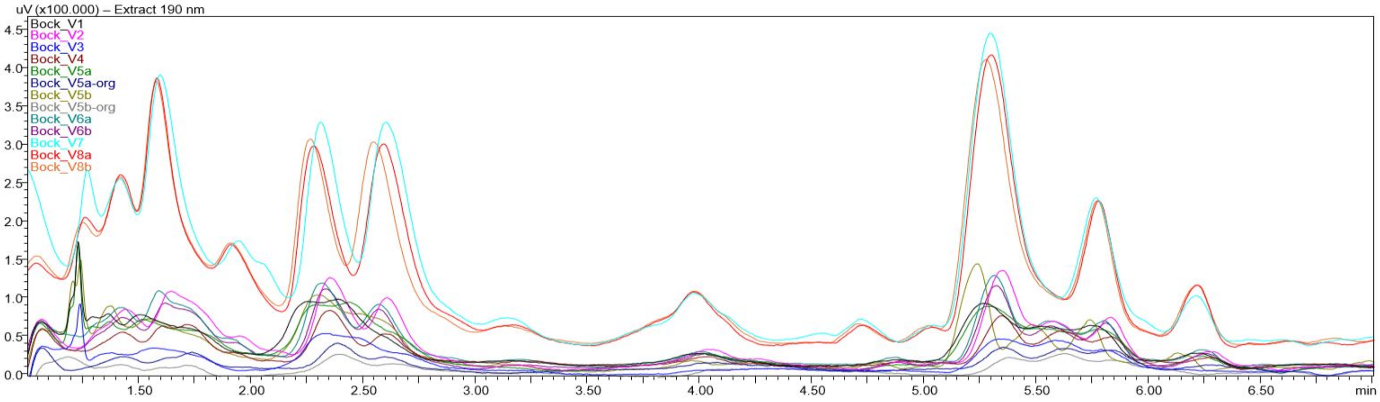

Figure S9. Extract of DAD UV at 190 nm for different sample preparation variations (bock beer).

2.2 LC-MS analyte signal area

To evaluate the reduction of matrix effects, the area of the peak was analyzed for all critical compounds. Here, the concentration was deliberately not calculated via SIDA as the A/IS ratio would compensate for losses or potential matrix effects. The following figures depict the signal area of the respective quantifier.

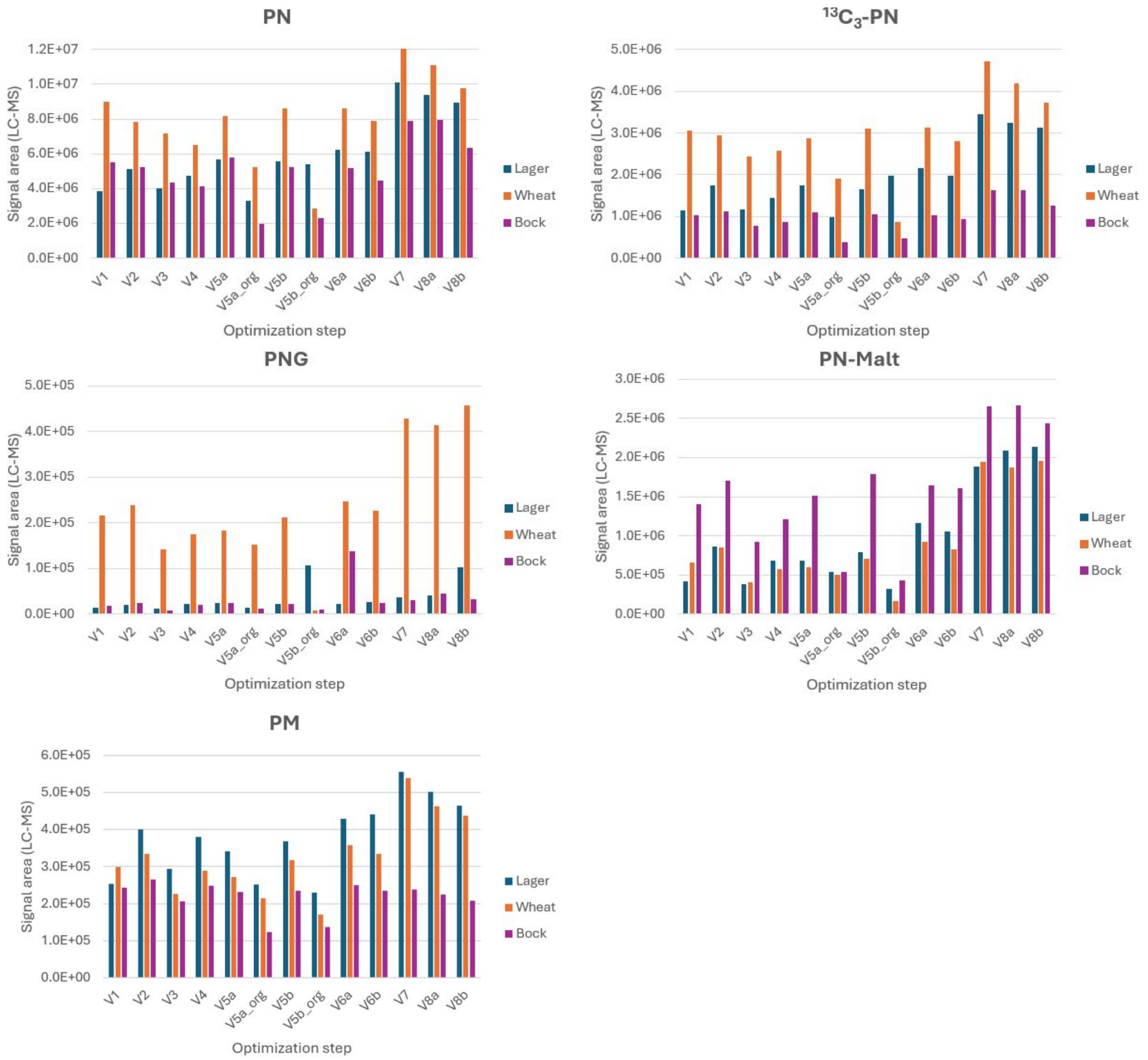

Figure S10. LC-MS peak area of the respective analytes considered for method optimization.

3. LC-MS/MS settings and results of the method validation

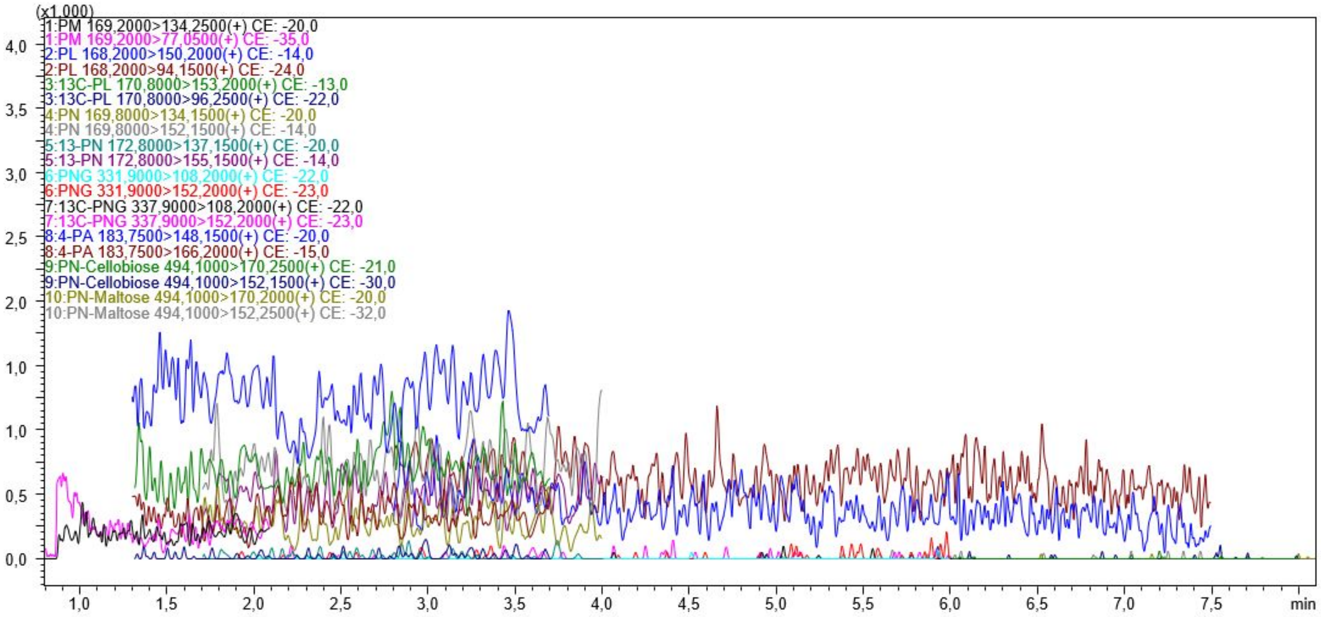

Figure S11. LC-MS/MS chromatogram of a reagent-blank sample.

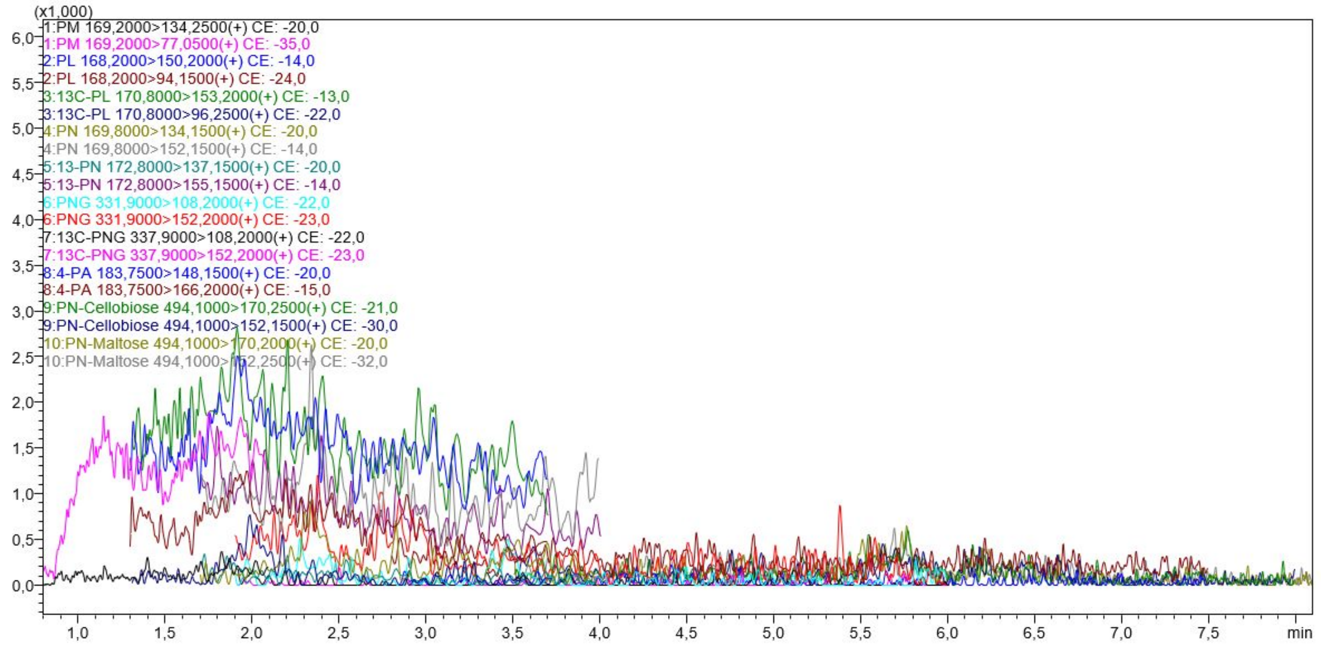

Figure S12. LC-MS/MS chromatogram of the artificial surrogate beer matrix.

Table S13. Precursor ions and product ions of the analytes and internal standards used for quantitation, with their respective optimized retention times (Rt) and fragmentation conditions.

**Analyte**	**Rt [min]**	**Precursor m/z**	**Product 1 [a] m/z**	**Product 2 [b] m/z**	**Dwell time [ms]**	**Voltage Q1 [V]**	**Collision Energy**	**Voltage Q3 [V]**
**PN**	2.34	169.80	134.15	152.15	13.0	-18.0	-20.0	-24.0
**$^{13}C_3$-PN**	2.33	172.80	137.15	155.15	19.0	-18.0	-20.0	-24.0
**PM**	1.03	169.20	134.25	77.05	26.0	-10.0	-20.0	-10.0
**PL**	1.76	168.20	150.20	94.15	19.0	-18.0	-14.0	-16.0
**$^{13}C_3$-PL**	1.76	170.80	153.20	96.25	13.0	-14.0	-13.0	-16.0
**4-PA**	4.43	183.75	148.15	166.20	16.0	-10.0	-15.0	-18.0
**PNG**	3.44	331.90	108.20	152.20	11.0	-14.0	-22.0	-22.0
**$^{13}C_6$-PNG**	3.04	337.90	108.20	152.20	11.0	-14.0	-22.0	-22.0
**PN-Malt**	6.17	494.10	170.20	152.25	16.0	-32.0	-20.0	-18.0
**PN-Cell**	6.42	494.10	170.25	152.15	16.0	-32.0	-21.0	-20.0

a: Quantifier.
b: Qualifier

Table S14. Internal standards, Regression equation, linear dynamic range, R^2-value (coefficient of determination), and LOD/LOQ.

**Analyte**	**Internal Std.**	**Response**	**Linear range**	**R²**	**LOD**	**LOQ**
			n(A)/n(IS)		[µg/L]	
**PN**	$^{13}C_3$-PN	y = 0.7626x - 0.0281	0.01-50.9	0.9996	0.27	1.06
**PM [a.b]**	$^{13}C_3$-PL	y = 1.0060x - 0.1358	0.02-24.9	0.9917	1.84	7.68
	$^{13}C_3$-PN	y = 01724x - 0.0257	0.01-10.2	0.9896		
**PL**	$^{13}C_3$-PL	y = 1.1794x + 0.0494	0.04-25.4	0.9976	2.94	12.9
**4-PA [a]**	$^{13}C_6$-PNG	y = 0.3988x - 0.0029	0.01-25.0	0.9996	1.26	4.87
**PNG**	$^{13}C_6$-PNG	y = 1.3190 + 0.0224	0.01-50.9	0.9993	0.07	0.31
**PN-Malt [a]**	$^{13}C_6$-PNG	y = 1.6560 - 0.0007	0.02-25.3	0.9999	0.08	0.31
**PN-Cell [a]**	$^{13}C_6$-PNG	y = 1.8259 + 0.0099	0.02-25.3	0.9999	0.05	0.20

a: Determined by matrix-matched calibration in the surrogate matrix with internal standard correlation.
b: Quantified by 13C3-PN in some beer samples.
PN, PL and PNG were determined by solvent calibration.

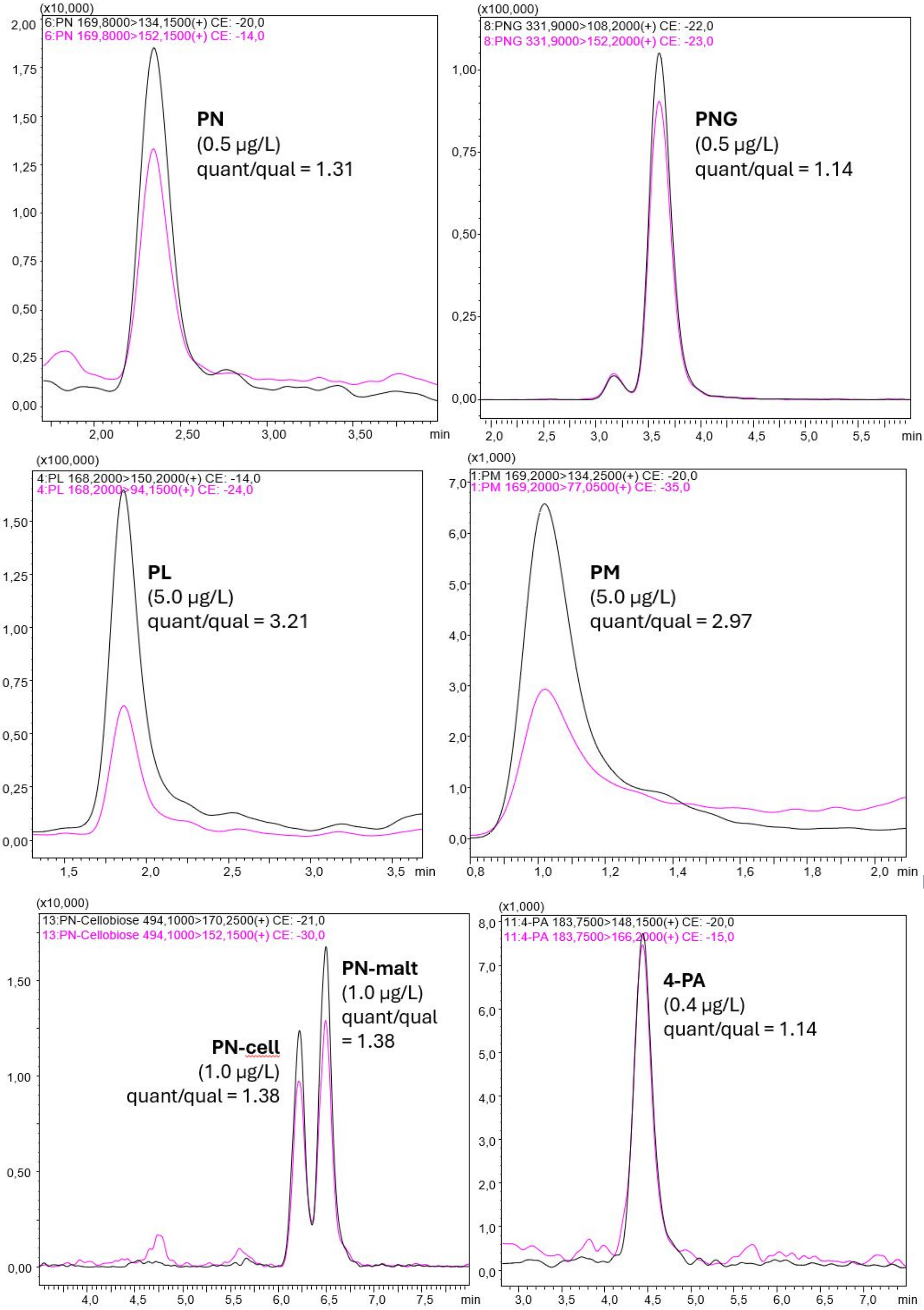

Figure

Figure S15. Chromatograms of B6 vitamers (quantifier and qualifier) at their respective lowest calibration levels.

PN/PNG: cal. level 1/100, c=0.5 µg/L; PL: cal. level 1/25, c=5.0 µg/L; PM: cal. level 1/100, c=5.0 µg/L; PN-cell/PN-malt: cal. level 1/25, c=1.0 µg/L; 4-PA: cal. level 1/100, c=0.4 µg/L.

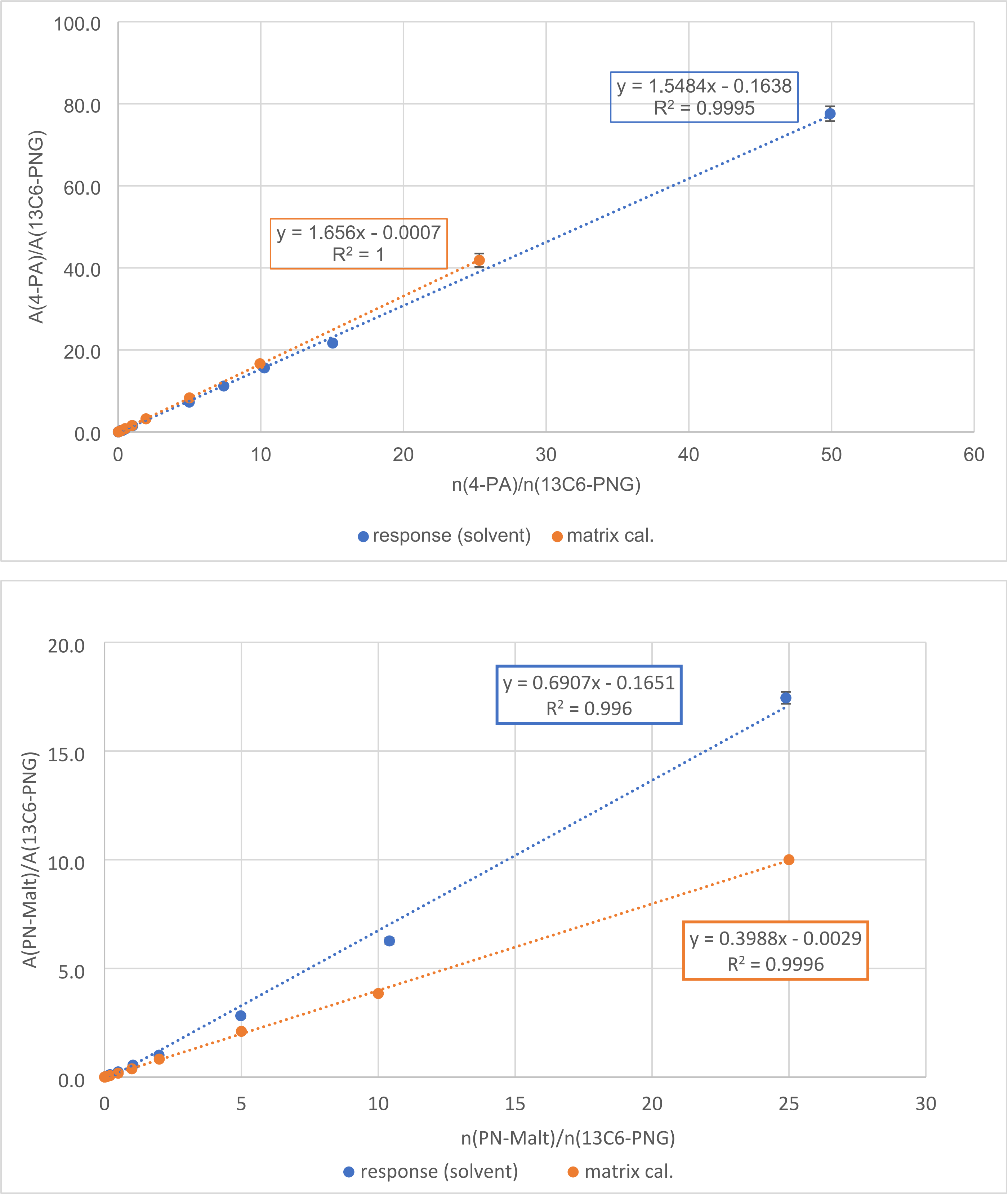

Figure S16. Comparison of response in solvent and surrogate matrix calibration for 4-PA (top) and PN-Malt (bottom).

Table S17: LOD/LOQ for all vitamin B6 analytes with uncertainties. Expanded uncertainties (u_{exp}) with (k = 2) were estimated at the LOD and LOQ concentrations by propagating concentration-dependent precision (u_{prec}), recovery bias (u_{bias}), and calibration slope uncertainty (u_{slope}).

Analyte	LOD					LOQ				
		u_{prec}	u_{bias}	u_{slope}	**u_{exp}**		u_{prec}	u_{bias}	u_{slope}	**u_{exp}**
					[µg/]					
**PN**	0.27	± 0.02	± 0.002	± 0.03	± 0.08	1.06	± 0.03	± 0.008	± 0.12	±0.25
**PM** [a.b]	1.84	± 0.19	± 0.01	± 1.63	± 3.29	7.68	± 0.21	± 0.06	± 6.82	± 13.7
**PL**	2.94	± 0.28	± 0.03	± 4.17	± 3.60	12.9	± 0.33	± 0.13	± 18.3	± 15.6
**4-PA** [a]	1.26	± 0.08	± 0.02	± 0.76	± 1.53	4.87	± 0.24	± 0.09	± 2.95	± 5.92
**PNG**	0.07	± 0.003	± 0.003	± 0.003	± 0.01	0.31	± 0.01	± 0.01	± 0.01	± 0.03
**PN-Malt** [a]	0.08	± 0.009	± 0.003	± 0.003	± 0.02	0.31	± 0.02	± 0.01	± 0.01	± 0.04
**PN-Cell** [a]	0.05	± 0.01	± 0.001	± 0.001	± 0.02	0.20	± 0.02	± 0.004	± 0.005	± 0.02

Table S18. Inter-injection, intra-day, and inter-day precisions given as relative standard deviation (RSD) for lager.

Analyte	Precision 1 (Lager)					
	Intra-day		*Inter-day*		*Inter-injection*	
	c ± SD [µg/L]	RSD [%]	c ± SD [µg/L]	RSD [%]	c ± SD [µg/L]	RSD. [%]
**PN**	378 ± 3.0	0.8	372.3 ± 4.5	1.3	374 ± 4.1	1.1
**PM** [a]	60.3 ± 0.8	1.3	59.9 ± 1.3	1.1	64.3 ± 1.5	2.3
**PL**	47.6 ± 0.4	0.8	46.6 ± 0.7	1.5	45.2 ± 1.1	2.3
**PNG**	36.7 ± 0.5	1.3	36.5 ± 0.5	1.4	35.9 ± 0.4	1.2
**PN-Malt**[a]	42.5 ± 0.7	1.7	42.5 ± 0.4	0.7	43.0 ± 0.8	1.8
**PN-Cell**[a]	5.1 ± 0.2	2.6	5.3 ± 0.1	3.7	374 ± 4.1	1.1

Table S19. Inter-injection, intra-day, and inter-day precisions given as relative standard deviation (RSD) for wheat beer.

Analyte	Precision 2 (Wheat Beer)					
	Intra-day		*Inter-day*		*Inter-injection*	
	c ± SD [µg/L]	RSD [%]	c ± SD [µg/L]	RSD [%]	c ± SD [µg/L]	RSD [%]
**PN**	268 ± 2.0	0.7	271 ± 3.8	1.4	272 ± 5.5	2.0
**PM** [a]	78.7 ± 4.0	5.1	76.3 ± 2.2	2.9	68.1 ± 3.4	5.0
**PL**	43.0 ± 0.6	1.5	42.0 ± 0.7	1.6	44.0 ± 1.1	2.5
**PNG**	61.2 ± 1.3	2.1	62.0 ± 1.1	1.8	61.2 ± 0.8	1.3
**PN-Malt**[a]	49.9 ± 0.9	1.8	51.2 ± 1.2	2.4	49.9 ± 0.6	1.3
**PN-Cell**[a]	16.8 ± 0.2	1.0	16.6 ± 0.2	1.5	16.4 ± 0.2	1.0

Table S20. Recoveries (Rec) of the single vitamers in surrogate matrix at four different concentration levels.

Analyte	Level 1			Level 2			Level 3			Level 4		
	c_{spike} [µg/L]	c_{found} ± SD [µg/L]	Rec [%]	c_{spike} [µg/L]	c_{found} ± SD [µg/L]	Rec [%]	c_{spike} [µg/L]	c_{found} ± SD [µg/L]	Rec [%]	c_{spike} [µg/L]	c_{found} ± SD [µg/L]	Rec [%]
**PN**	10.0	10.3 ± 0.10	103	100	101 ± 0.73	101	500	473 ± 1.55	95.0	1000	995 ± 18.0	99.5
**PM** [a]	7.50	7.38 ± 0.09	98.4	20.0	19.7 ± 0.69	98.5	50.0	49.9 ± 2.09	100	100	105 ± 4.54	105
**PL**	10.0	10.3 ± 0.18	102	40.0	39.5 ± 1.24	98.8	50.0	50.5 ± 0.65	101	100	102 ± 0.89	102
**4-PA** [a]	7.00	7.16 ± 0.42	103	10.0	9.13 ± 0.34	91.3	50.0	42.9 ± 1.84	85.8	100	82.7 ± 2.22	82.7
**PNG**	2.00	2.04 ± 0.07	103	10.0	9.70 ± 0.00	97.0	100	98.4 ± 2.39	98.4	200	203 ± 5.85	101
**PN-Malt**[a]	2.00	2.02 ± 0.03	101	50.0	48.3 ± 0.50	96.5	200	193 ± 5.51	96.6	500	485 ± 24.5	97.0
**PN-Cell**[a]	2.00	2.05 ± 0.00	103	50.0	10.3 ± 0.09	103	50.0	49.3 ± 0.40	98.5	100	106 ± 0.24	106

Table S21. Recoveries (Rec) of the single vitamers in surrogate matrix at four concentration levels used for calculating LOD and LOQ with standard deviation (SD) and relative standard deviation (RSD).

Analyte	Concentration level 1				Concentration level 2				Concentration level 3				Concentration level 4			
	c_{spike} [µg/L]	c_{found} ± SD [µg/L]	RSD [%]	Rec [%]	c_{spike} [µg/L]	c_{found} ± SD [µg/L]	RSD [%]	Rec [%]	c_{spike} [µg/L]	c_{found} ± SD [µg/L]	RSD [%]	Rec [%]	c_{spike} [µg/L]	c_{found} ± SD [µg/L]	RSD [%]	Rec [%]
**PN**	1.00	1.15 ± 0.02	1.4	115	4.00	4.06 ± 0.10	2.4	102	7.00	7.05 ± 0.14	2.0	101	10.0	10.3 ± 0.10	1.0	103
**PM** [a.b]	7.50	7.38 ± 0.09	1.3	98.4	30.0	30.4 ± 1.10	3.6	101	52.5	52.1 ± 0.41	0.79	99.2	75.0	76.4 ± 0.90	1.2	102
**PL**	10.0	10.3 ± 0.18	1.8	102	40.0	39.5 ± 1.24	3.2	98.8	70.0	71.6 ± 1.38	1.9	102	100.0	105 ± 1.19	1.1	105
**4-PA** [a]	0.75	0.73 ± 0.02	3.3	97.4	3.00	2.81 ± 0.08	2.8	93.5	52.5	4.36 ± 0.81	19	83.1	7.00	7.16 ± 0.42	5.9	103
**PNG**	0.20	0.21 ± 0.01	3.3	103	0.80	0.80 ± 0.01	1.5	100	1.40	1.42 ± 0.04	2.6	101	2.00	2.04 ± 0.07	3.6	102
**PN-Malt**[a]	0.20	0.20 ± 0.01	6.3	102	0.80	0.81 ± 0.02	2.5	101	1.40	1.38 ± 0.07	4.8	98.6	2.00	2.02 ± 0.03	1.6	101
**PN-Cell**[a]	0.20	0.20 ± 0.01	3.2	102	0.80	0.81 ± 0.02	2.9	102	1.40	1.44 ± 0.05	3.4	103	2.00	2.05 ± 0.00	0.2	103

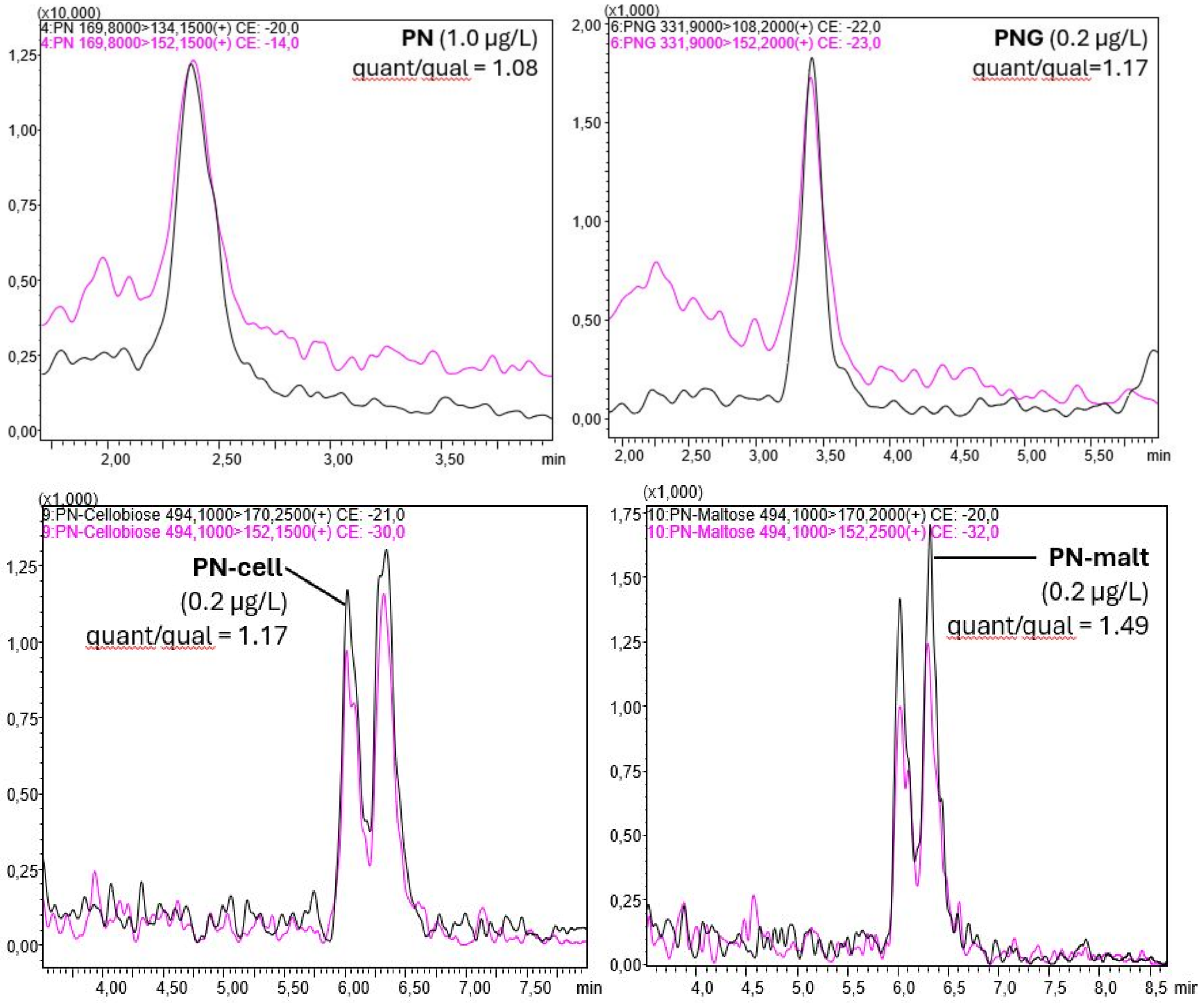

Figure S22. Chromatograms of quantifier (quant) and qualifier (qual) transitions of analytes with low LODs at LOD level in surrogate matrix.

Table S23. Quantifier/qualifier ratios ± SD and RSD in brackets ratios of all analytes in different matrices.

	**Quantifier/Qualifier Ratio**			
**Analyte**	**Response (solvent)**	**Surrogate Matrix**	**Concentration levels 1-4 for LOD determination (cf S21)**	**Beer samples**
**PN**	1.17 ± 0.22 (19%)	1.09 ± 0.01 (0.7%)	1.18 ± 0.12 (9.1%)	1.17 ± 0.12 (10%)
**$^{13}C_3$-PN**	1.24 ± 0.03 (2.6%)	1.16 ± 0.08 (6.7%)	1.22 ± 0.03 (2.1%)	1.21 ± 0.14 (12%)
**PM** [a.b]	2.98 ±0.12 (4.2%)	3.03 ± 0.39 (13%)	3.24 ± 0.35 (11%)	2.95 ± 0.47 (16%)
**PL**	2.84 ± 0.39 (14%)	2.72 ± 0.34 (12%)	2.70 ± 0.32 (12%)	2.58 ± 0.42 (16%)
**$^{13}C_3$-PL**	2.43 ± 0.69 (2.8%)	2.35 ± 0.12 (5.2%)	2.41 ± 0.21 (8.8%)	2.45 ± 0.33 (14%)
**4-PA** [a]	1.02 ± 0.14 (14%)	1.18 ± 0.09 (7.1%)	1.12 ± 0.07 (6.1%)	1.21 ± 0.10 (8.7%)
**PNG**	1.37 ± 0.08 (6.1%)	1.32 ± 0.06 (4.7%)	1.31 ± 0.10 (7.7%)	1.31 ± 0.10 (7.8%)
**$^{13}C_6$-PNG**	1.40 ± 0.21 (15%)	1.34 ± 0.09 (6.7%)	1.35 ± 0.07 (5.4%)	1.27 ± 0.09 (6.7%)
**PN-Malt** [a]	1.50 ± 0.09 (6.0%)	1.39 ± 0.04 (3.0%)	1.39 ± 0.14 (10%)	1.30 ± 0.18 (6.5%)
**PN-Cell** [a]	1.51 ± 0.16 (10%)	1.26 ± 0.08 (6.4%)	1.32 ± 0.13 (9.7%)	1.35 ± 0.11 (7.8%)

4. Summary: Vitamin B6 content in beers

Table S24. Individual vitamer concentration and total B6 concentration of all beer samples calculated as PN-eq. in µg/L. SD Standard deviation of total B6 content. AF alcohol free.
For alcohol-free beer: # sugar content <2.0 g/100g; * sugar content ≥2.0 g/100g.

Beer	PN	PNG	PNG in PN eq.	PN-Malt	Malt in PN eq.	PN-Cell	Cell in PN eq.	PM	PL	Total B6	SD
					[µg/L]						
B	729	34.6	17.7	114	39.2	n/d	n/d	61.2	12.6	860	44.2
	721	13.6	7.0	160	54.9	n/d	n/d	74.2	n/d	857	15.0
	697	44.3	22.6	118	40.5	n/d	n/d	70.1	n/d	831	16.4
	624	0.97	0.5	123	42.1	n/d	n/d	62.3	n/d	729	62.1
	449	n/d	n/d	47.4	16.2	6.86	2.35	63.1	n/d	531	28.7
	578	3.26	1.67	32.0	11.0	5.18	1.77	77.3	15.9	686	11.6
	879	14.8	7.58	122	42.0	5.74	1.97	67.3	18.5	1020	122
	666	16.1	8.22	44.1	15.1	6.14	2.11	36.2	105	832	64.3
	464	5.64	2.88	27.2	9.31	5.48	1.88	49.0	59.8	594	17.3
D	574	7.83	4.00	75.9	26.0	n/d	n/d	59.2	4.96	668	85.8
	463	31.6	16.1	92.3	31.6	n/d	n/d	54.4	8.30	574	10.8
	394	41.8	21.4	46.8	16.1	n/d	n/d	72.5	n/d	504	20.2
	534	4.42	2.26	25.1	8.60	n/d	n/d	68.2	n/d	613	33.7
	481	61.2	31.3	35.0	12.0	3.64	1.25	43.9	17.0	587	11.1
L	525	11.5	5.86	40.2	13.8	n/d	n/d	62.8	37.8	645	24.7
	374	36.8	18.8	42.4	14.5	n/d	n/d	66.0	45.7	519	4.11
	295	25.2	12.9	15.2	3.48	11.5	3.95	74.5	n/d	389	64.9
	346	0.73	0.37	47.2	16.2	n/d	n/d	71.3	n/d	434	5.84
	391	n/d	n/d	36.7	12.6	11.7	4.03	82.4	n/d	490	18.5
	312	29.6	15.1	27.6	9.47	11.1	3.80	104	n/d	444	9.58
	446	0.56	0.28	47.9	16.4	11.5	3.93	84.7	n/d	552	65.5
	401	11.6	5.92	26.3	9.01	n/d	n/d	102	n/d	518	40.6
	456	11.8	6.02	37.8	12.9	5.58	1.91	27.6	64.2	568	0.69
	486	9.03	4.61	30.4	10.4	5.42	1.86	32.9	54.8	591	30.3
L af #	571	17.3	8.81	80.1	27.5	12.9	4.44	99.4	50.0	762	17.4
	400	31.8	16.2	55.8	19.1	8.80	3.02	36.7	43.1	519	2.16
	316	n/d	11.9	63.7	21.9	10.4	3.56	36.3	70.3	460	10.9
	533	5.17	2.64	37.3	12.8	n/d	n/d	32.2	83.8	664	6.20
	404	31.3	16.0	53.8	18.4	n/d	n/d	33.7	54.5	526	3.49
L af *	178	126	64.4	32.3	11.1	8.56	2.93	48.7	n/d	305	7.11
	206	103	52.7	18.7	6.41	n/d	n/d	21.9	11.3	298	1.38
	213	85.1	43.4	28.8	9.87	3.73	1.28	27.8	37.5	333	99.5
	224	157	80.3	41.1	14.1	n/d	n/d	24.8	12.0	355	7.39
	273	149	76.3	70.3	24.1	n/d	n/d	23.7	18.6	416	10.7
	245	140	71.5	22.7	7.78	n/d	n/d	22.2	7.13	354	16.9

Table S24. *(continuation)* Individual vitamer concentration and total B_6 concentration of all beer samples calculated as PN-eq. in µg/L. SD Standard deviation of total B_6 content. AF alcohol free. For alcohol-free beer: # sugar content <2.0 g/100g; * sugar content ≥2.0 g/100g.

Beer	PN	PNG	PNG in PN eq.	PN-Malt	Malt in PN eq.	PN-Cell	Cell in PN eq.	PM	PL	Total B6	STD
					[µg/L]						
**P**	364	14.1	7.22	53.8	18.4	n/d	n/d	71.5	39.7	501	4.16
	474	8.26	4.22	54.5	18.7	n/d	n/d	82.2	42.0	621	20.3
	229	28.9	14.7	42.4	14.5	n/d	n/d	82.1	n/d	340	13.7
	449	4.67	2.38	29.6	10.2	n/d	n/d	85.8	30.2	578	7.78
	462	33.2	17.0	36.2	12.4	n/d	n/d	29.3	54.0	575	17.5
**R**	130	48.1	24.6	44.8	15.4	12.7	4.35	26.6	25.5	247	38.2
	158	18.0	9.20	25.0	8.58	n/d	n/d	30.4	38.4	213	35.6
	28.9	31.8	16.2	24.8	8.50	n/d	n/d	24.4	17.4	106	22.9
**U**	357	41.5	21.2	53.5	18.4	n/d	n/d	75.7	27.6	500	29.4
	553	56.9	29.0	84.0	28.8	n/d	n/d	67.6	19.1	697	33.9
	311	0.38	0.19	36.9	12.6	6.63	2.27	81.6	n/d	408	50.8
	405	7.60	3.88	52.4	18.0	13.2	4.52	81.0	n/d	512	12.0
	363	0.76	0.39	24.3	8.32	n/d	n/d	108	n/d	480	4.40
	372	53.1	27.1	43.7	15.0	n/d	n/d	94.1	n/d	508	17.6
	553	28.0	14.3	61.9	21.2	6.48	2.22	30.3	55.6	677	12.5
**WB**	397	7.77	3.97	33.9	11.6	16.1	5.51	65.8	26.7	511	16.9
	263	42.6	21.8	48.2	16.5	17.4	5.96	64.9	38.7	411	26.8
	194	23.6	12.1	51.5	17.6	19.8	6.80	58.0	11.2	300	10.2
	320	8.04	4.11	32.5	11.1	10.0	3.43	59.9	15.2	413	22.6
	148	40.8	20.8	48.2	16.5	21.7	7.44	63.5	n/d	256	12.1
	165	28.3	14.5	24.8	8.51	20.6	7.07	61.9	n/d	257	3.65
	203	1.43	0.73	52.4	18.0	17.6	6.02	80.0	n/d	307	13.5
	308	7.51	3.83	23.4	8.02	8.49	2.91	29.3	55.2	407	12.8
	340	6.85	3.50	24.4	8.36	9.17	3.14	29.0	37.7	421	1.34
	312	11.5	5.88	25.9	8.89	8.30	2.85	28.1	47.1	405	23.7
	383	6.19	3.16	27.0	9.24	9.73	3.34	44.1	17.9	461	19.5
**WB af #**	148	44.7	22.8	45.9	15.7	21.9	7.52	49.1	n/d	243	0.66
	360	7.12	3.64	52.4	18.0	18.4	6.31	38.7	23.2	450	6.49
	151	20.0	10.2	45.0	15.4	18.5	6.33	26.0	24.2	233	3.01
	401	7.76	3.96	29.1	10.0	9.71	3.33	30.4	50.0	498	52.8
**WB af ***	191	52.6	26.8	30.7	10.5	12.9	4.43	47.5	n/d	280	2.18
	249	72.5	37.0	59.5	20.4	23.0	7.87	48.4	n/d	362	20.2
	227	96.8	49.4	20.7	7.11	11.0	3.76	42.6	n/d	329	9.48
	360	7.1	3.64	52.4	18.0	18.4	6.31	38.7	23.2	450	6.49

5. Literature

(1) Back, D. F.; de Oliveira, G. M.; Lang, E. S., Chelation of UO22+ by vitamin B6 complex derivatives: Synthesis and characterization of [UO2(β-pyracinide)2(H2O)] and [UO2(Pyr2en)DMSO]Cl2{Pyr2en=N,N′-ethylenebis(pyridoxylideneiminato)}. A useful modeling of assimilation of uranium by living beings. *Journal of Inorganic Biochemistry* **2006,** *100* (10), 1698–1704, DOI: https://doi.org/10.1016/j.jinorgbio.2006.06.004.
